# Distinct and Atypical Intrinsic and Extrinsic Cell Death Pathways between Photoreceptor Cell Types upon Specific Ablation of *Ranbp2* in Cone Photoreceptors

**DOI:** 10.1371/journal.pgen.1003555

**Published:** 2013-06-20

**Authors:** Kyoung-in Cho, MdEmdadul Haque, Jessica Wang, Minzhong Yu, Ying Hao, Sunny Qiu, Indulekha C. L. Pillai, Neal S. Peachey, Paulo A. Ferreira

**Affiliations:** 1 Department of Ophthalmology, Duke University Medical Center, Durham, North Carolina, United States of America; 2 Department of Ophthalmic Research, Cole Eye Institute, Cleveland Clinic Foundation, Cleveland, Ohio, United States of America; 3 Research Service, Cleveland Veterans Affairs Medical Center, Cleveland, Ohio, United States of America; 4 Department of Ophthalmology, Cleveland Clinic Lerner College of Medicine of Case Western Reserve University, Cleveland, Ohio, United States of America; 5 Department of Pathology, Duke University Medical Center, Durham, North Carolina, United States of America; University of Minnesota, United States of America

## Abstract

Non-autonomous cell-death is a cardinal feature of the disintegration of neural networks in neurodegenerative diseases, but the molecular bases of this process are poorly understood. The neural retina comprises a mosaic of rod and cone photoreceptors. Cone and rod photoreceptors degenerate upon rod-specific expression of heterogeneous mutations in functionally distinct genes, whereas cone-specific mutations are thought to cause only cone demise. Here we show that conditional ablation in cone photoreceptors of *Ran-binding protein-2* (*Ranbp2*), a cell context-dependent pleiotropic protein linked to neuroprotection, familial necrotic encephalopathies, acute transverse myelitis and tumor-suppression, promotes early electrophysiological deficits, subcellular erosive destruction and non-apoptotic death of cones, whereas rod photoreceptors undergo cone-dependent non-autonomous apoptosis. Cone-specific *Ranbp2* ablation causes the temporal activation of a cone-intrinsic molecular cascade highlighted by the early activation of metalloproteinase 11/stromelysin-3 and up-regulation of *Crx* and *CoREST*, followed by the down-modulation of cone-specific phototransduction genes, transient up-regulation of regulatory/survival genes and activation of caspase-7 without apoptosis. Conversely, PARP1^+^-apoptotic rods develop upon sequential activation of caspase-9 and caspase-3 and loss of membrane permeability. Rod photoreceptor demise ceases upon cone degeneration. These findings reveal novel roles of *Ranbp2* in the modulation of intrinsic and extrinsic cell death mechanisms and pathways. They also unveil a novel spatiotemporal paradigm of progression of neurodegeneration upon cell-specific genetic damage whereby a cone to rod non-autonomous death pathway with intrinsically distinct cell-type death manifestations is triggered by cell-specific loss of *Ranbp2*. Finally, this study casts new light onto cell-death mechanisms that may be shared by human dystrophies with distinct retinal spatial signatures as well as with other etiologically distinct neurodegenerative disorders.

## Author Summary

The secondary demise of healthy neurons upon the degeneration of neurons harboring primary genetic defect(s) is hallmark to neurodegenerative diseases. However, the factors and mechanisms driving these cell-death processes are not understood, a severe limitation which has hampered the therapeutic development of neuroprotective approaches. The neuroretina is comprised of two main types of photoreceptor neurons, rods and cones. These undergo degeneration upon heterogeneous mutations or environmental stressors and the underlying diseases present conspicuous spatiotemporal pathological signatures whose molecular bases are not understood. We employed the multifunctional protein, Ran-binding protein-2 (Ranbp2), which is implicated in cell-type and stress-dependent clinical manifestations, to examine its role(s) in primary and secondary photoreceptor death mechanisms upon its specific loss in cones. Contrary to prior findings, we found that dying cones can trigger the loss of healthy rods. This process arises by the immediate activation of novel Ranbp2-responsive factors and downstream cascade events in cones that promote extrinsically the demise of rods. The mechanisms of rod and cone demise are molecularly distinct. Collectively, the data uncover distinct Ranbp2 roles in intrinsic and extrinsic cell-death and will likely contribute to our understanding of the spatiotemporal onset and progression of diseases affecting photoreceptor mosaics and other neural networks.

## Introduction

The disintegration of neuronal networks owing to the non-autonomous death of neurons without primary damage is a hallmark manifestation of many neurodegenerative diseases and contributes determinately to their onset or progression [1]–[3]. Cone or rod photoreceptor neurons employ cell type-specific spectrally tuned and highly homologous phototransduction cascades. Neurodegenerative disorders affecting these neurons serve as excellent models to understand autonomous and non-autonomous cell death processes. First, early studies with chimeric mice with a mixture of healthy and unhealthy rod photoreceptors owing to the expression of rod-specific degenerative mutations by the latter showed that damaged rod photoreceptors promote the non-autonomous death of healthy rod photoreceptors [4]–[7], but the analogous event does not appear to occur between neighboring healthy and damaged cone photoreceptors [8]. Second, rod photoreceptor-specific mutations causing the death of rod photoreceptors promote ultimately the non-autonomous death of cone photoreceptors [3], [9]–[12]. This secondary loss of cone photoreceptors has the greatest impact on human vision, because cone photoreceptors mediate daylight and high acuity vision as well as color perception. For example, rod photoreceptor-specific mutations affecting phototransduction components of rod photoreceptors, such as the catalytic subunit of cGMP phosphodiesterase, rhodopsin and cyclic nucleotide-gated (CNG) channel subunits, lead to the degeneration of damaged rod and healthy cone photoreceptors [4], [9], [11]–[16]. By contrast, the cellular effects of cone photoreceptor-specific mutations causing cone degeneration are much less clear, but they are thought to spare the viability of rod photoreceptors. For example, cone-specific mutations impairing genes homologous to those of rod phototransduction cause the death of cone photoreceptors only [17]–[26].

A number of distinct models have been put forward to explain the secondary loss of healthy cone photoreceptors upon the primary degeneration of damaged rod photoreceptors. These include oxidative stress and metabolic imbalance caused by increased oxygen tension [27]–[29], loss of paracrine neurotrophic [30] or vasculotrophic support [31], microglia activation [32], [33] and release of rod-derived toxic byproducts [34]. Distinguishing what mechanisms trigger extrinsic-elicited cell death pathways is further complicated by our limited knowledge of the intrinsic and primary cell-death pathways affecting rod photoreceptor themselves, and whether these are consistent across rod degeneration models [35]–[45]. Likewise, the knowledge about the molecular and subcellular events underlying the primary demise of cone photoreceptors is very limited [19], [46]. Ascertaining the spatiotemporal processes causing autonomous and non-autonomous neural death is critical to our understanding of the pathogenesis of a variety of human photoreceptor dystrophies, such as retinitis pigmentosa (RP) and age-related macular degeneration (AMD), that harbor hallmark spatiotemporal manifestations presumably driven by distinct intrinsic and extrinsic factors.

The pleiotropic protein, Ran-binding protein-2 (RanBP2), is essential for organism viability and energy metabolism [47], [48]. Prior studies on RanBP2 indicate that it plays critical cell-type-dependent physiological roles in mediating gene-environment interactions. In this regard, distinct disease stressors, such as phototoxicity [49], [50], Parkinsonian toxic insults [51], and carcinogens [48], trigger a variety of cell-context-dependent clinical and pathophysiological manifestations upon partial deficits or mutations of *Ranbp2*. Further, semi-dominant mutations in human *RANBP2* cause either acute necrotizing encephalopathies (ANE1) or acute transverse myelitis (ATM) upon exposure to a variety of infectious agents [52]–[54]. In this study we set out to determine the intrinsic and extrinsic effects of lack of Ranbp2 function in the survival of cone or rod photoreceptor neurons upon selective ablation of *Ranbp2* in cone photoreceptors, where Ranbp2 is highly expressed [55]. We show that cone-specific ablation of *Ranbp2* promotes the autonomous non-apoptotic death of cone photoreceptors and the cone-dependent apoptotic demise of rod photoreceptors by distinct cell-type death mechanisms. Hence, a primary impairment of cone photoreceptors can promote the secondary death of healthy rod photoreceptors, a paradigm-shift observation with implications to our understanding of human neurodegenerative diseases affecting distinct photoreceptor cell types with hallmark regional distributions in the retina and other neural networks of the central nervous system.

## Results

### Generation of mice with ablation of *Ranbp2* selectively in cone photoreceptors

**Figure 1:**
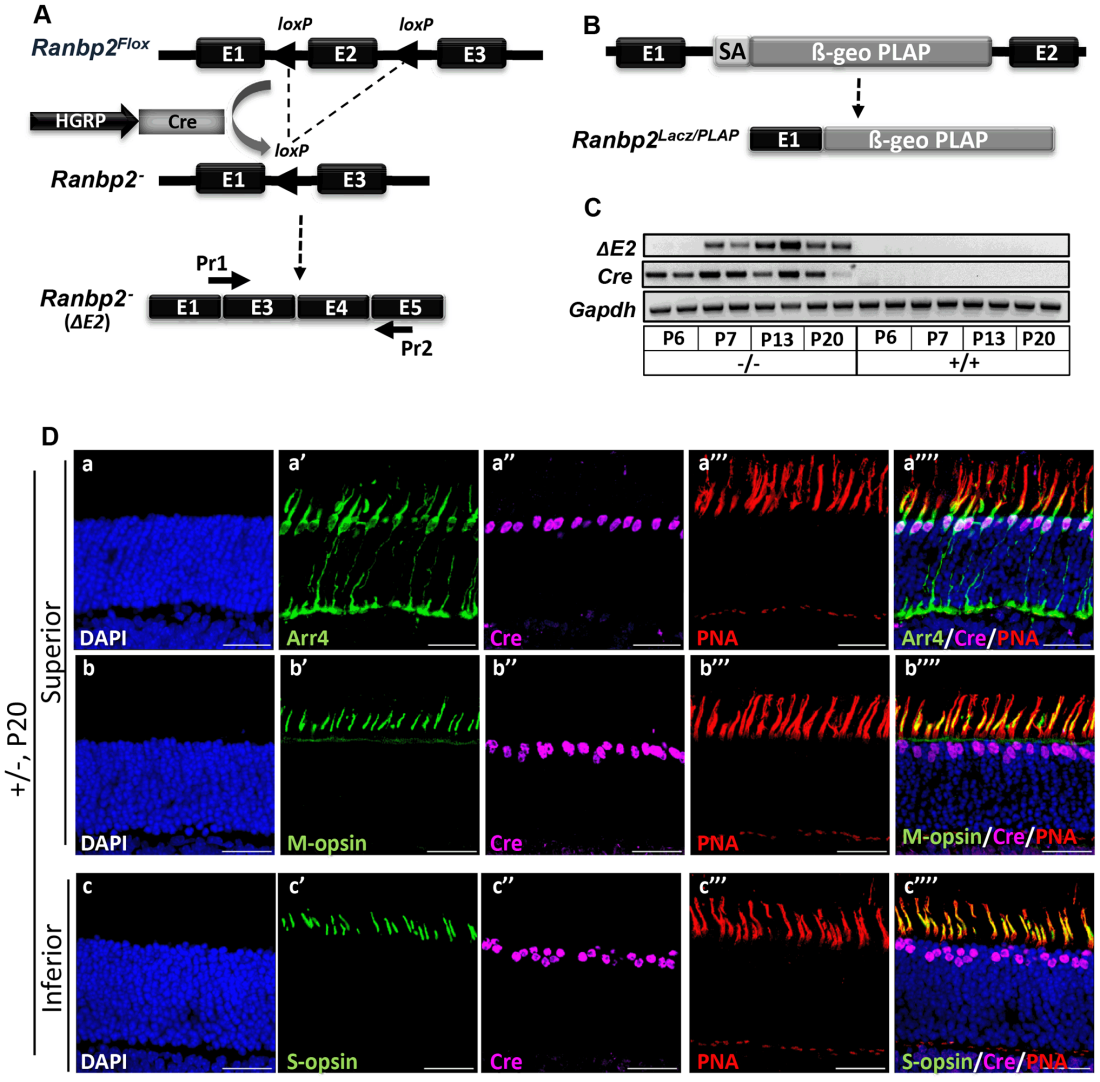
Cre-mediated ablation of *Ranbp2* selectively in cone photoreceptors. (**A**) Schematic diagram for targeted *Ranbp2* allele upon HGRP-driven Cre excision of exon 2 and production of the recombinant mRNA of *Ranbp2.* Pr1 and Pr2 are specific to fused exons 1 and 3, and exon 5, respectively, and were used to monitor the functional excision of *Ranbp2^Flox^* allele. (**B**) Schematic diagram of a constitutively targeted *Ranbp2* allele upon insertion of a promoterless bicistronic (β-geo-PLAP) cassette with a splicing acceptor site (SA) between exons 1 and 2. (**C**) RT-PCR of recombinant *Ranbp2* mRNA without exon 2 using Pr1 and Pr2 primers. Excision of exon 2 (***Δ***E2) was detectable in *HRGP-cre:Ranbp2^−/−^* (−/−) but not wild-type (+/+) mice at P7 of age, a day after expression of *Cre* recombinase. (**D**) Co-expression (a″″–c″″) of Arr4 (a′), M-opsin (b′), S-opsin (c′), Cre (a″–c″) and PNA (a′″–c′″) in cone photoreceptor neurons of the superior (dorsal, a–b″″) or inferior (ventral, c–c″″) regions of the retina of *HRGP-cre:Ranbp2^+/−^* mice at P15. Sections were counterstained with DAPI (a–c). Legends: HGRP, L/M opsin promoter; Arr4, cone arrestin 4; PNA, Peanut Agglutinin. Scale bars = 25 µm.

To determine the physiological role of *Ranbp2* in cone photoreceptors and uncover the effects of its genetic ablation in autonomous and non-autonomous molecular and cellular events affecting targeted cones and healthy rod photoreceptors, *Ranbp2* was selectively targeted in mouse cones by Cre-mediated recombination of floxed *Ranbp2* [48], [56], [57] (Figure 1A). Hemizygous transgenic mice expressing Cre under control of the R/G cone opsin promoter (*Tg-HRGP-cre*) [56], [57] were crossed with *Ranbp2^Flox/+^* mice [48] to produce *Tg-HRGP-cre:Ranbp2^Flox/+^*. These mice were then crossed to *Ranbp2^Flox/+^* or *Ranbp2^Gt(pGT0pfs)630Wcs/+^* , which harbors a constitutively disrupted allele of *Ranbp2* [47] (Figure 1B), to generate the lines, *Tg-HRGP-cre:Ranbp2^Flox/Flox^* and *Tg-HRGP-cre:Ranbp2^Flox/Gt(pGT0pfs)630Wc^*. These lines were morphologically and functionally indistinguishable from each other and they are hereafter designated as *HRGP-cre:Ranbp2^−/−^*. An out-of-frame *Ranbp2* transcript comprising the fusion of exons 1 and 3 is produced at P7 upon Cre expression at P6 (Figure 1C). Cre was specifically expressed in cell bodies of M- and S-cone photoreceptors (Figure 1D, Figure S1).

### *HRGP-cre:Ranbp2^−/−^* mice present rampant degeneration of cone photoreceptors

**Figure 2:**
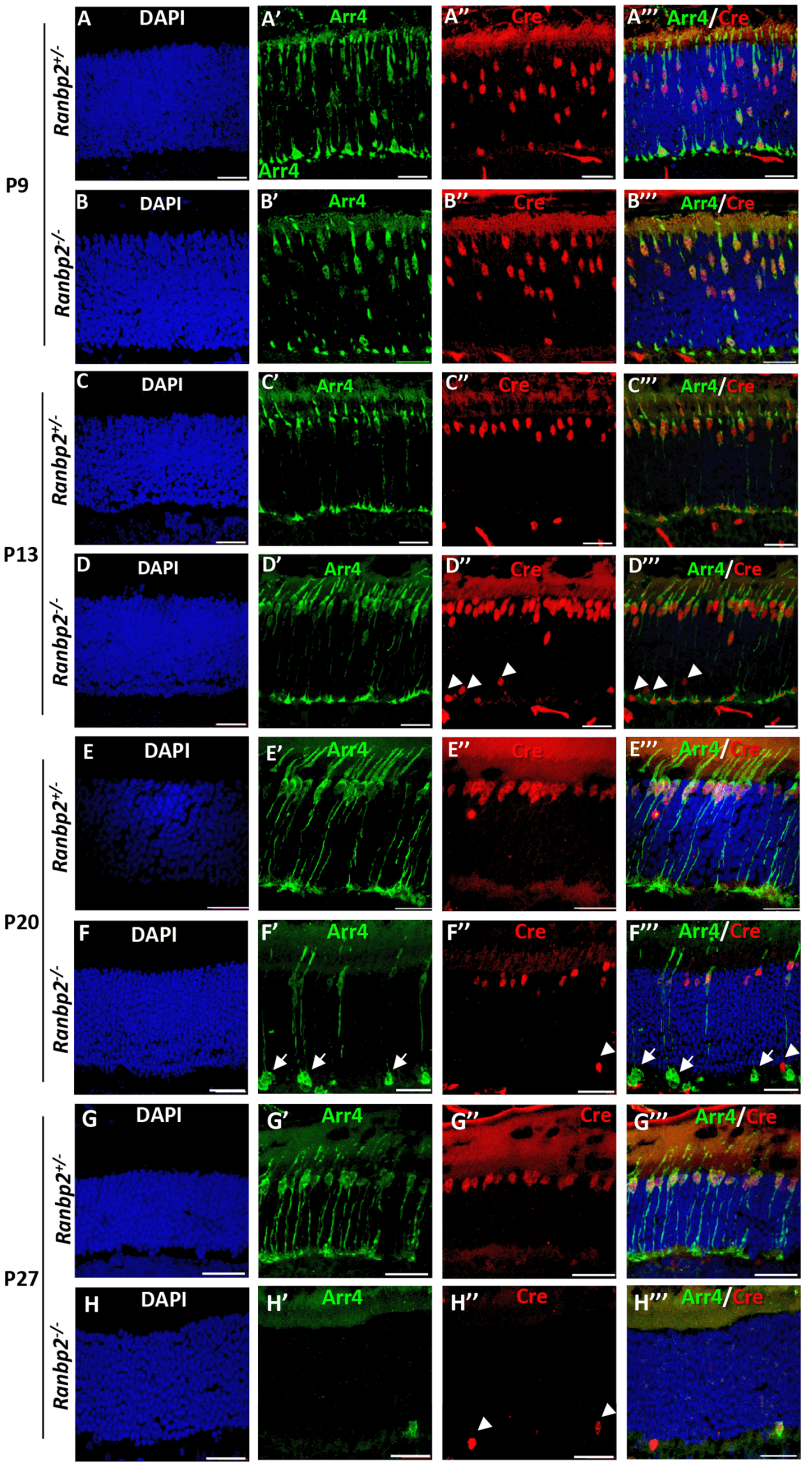
Temporal and morphological profile of degeneration of cone photoreceptors in *Ranbp2^−/−^* mice. (**A–H**′″) Immunohistochemistry of retinal sections of *HRGP-cre:Ranbp2^+/−^* (*Ranbp2^+/−^*) and *HRGP-cre:Ranbp2^−/−^* (*Ranbp2^−/−^*) mice at P9 (**A–B**′″), P13 (**C–D**′″) P20 (**E–F**′″) and P27 (**G–H**′″) of age with the antibodies against Cre recombinase and cone arrestin (Arr4) showing rapid loss of cone photoreceptors in *RanBP2^−/−^*. Cre co-localized with Arr4 only in DAPI-stained cell bodies of cone cells throughout all ages regardless of genotype. Arrowheads in indicate Cre^+^ nuclei retracted to the proximal ONL (outer nuclear layer) in *Ranbp2^−/−^* at P13, P20 and P27 of age, respectively. Arrows point to prominent swellings of synaptic pedicles of cone photoreceptors in *Ranbp2^−/−^* at P20 of age. Scale bars = 25 µm.

We examined the temporal effects of loss of *Ranbp2* expression in the morphology and survival of cones by comparing the immunostaining of retinal sections between *HRGP-cre:Ranbp2^−/−^* and *HRGP-cre:Ranbp2^+/−^* mice with anti-Cre and cone-specific anti-arrestin-4 (Arr4) antibodies [58] at P9, P13, P20 and P27 of age (Figure 2). In both genotypes, the cell bodies of Cre-expressing cone photoreceptors migrated to the distal (outer) region of the outer nuclear layer (ONL) by P13, where the majority of cone cell bodies are typically localized, and the outer segment (OS) and synaptic pedicles developed properly. However, few Cre^+^-cell bodies appeared displaced in the proximal (inner) ONL of *HRGP-cre:Ranbp2^−/−^* mice (Figure 2D″). By P20, cones of *HRGP-cre:Ranbp2^−/−^* mice presented prominent swelling of the synaptic pedicles (Figure 2F′) and retraction of some cell bodies to the proximal region of the ONL (Figure 2F″). By P27, only a very few surviving cones were present in *HRGP-cre:Ranbp2^−/−^* (Figure 2H′–2H″); by 6 weeks resilient cones lacking outer segments were rarely present (Figure S2), whereas no cones were present in 12-week old mice (data not shown).

**Figure 3:**
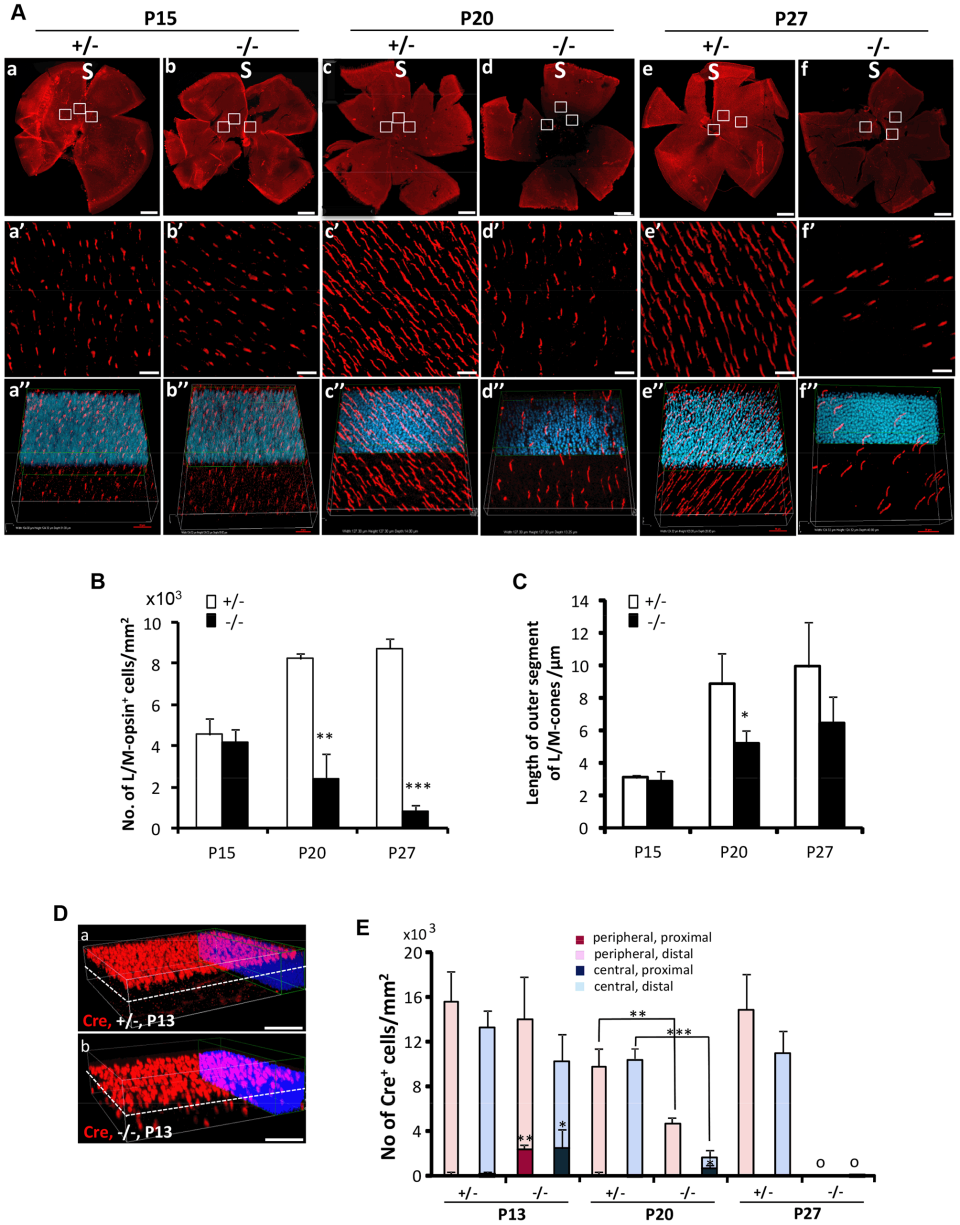
Topographic degeneration of M-cone photoreceptors and their outer segments in *HRGP-cre:Ranbp2^−/−^*. (**A**) Retinal flat mount images of *HRGP-cre:Ranbp2^−/−^* (−/−) and *HRGP-cre:Ranbp2^+/−^* (+/−) immunostained with an antibody against M-opsin showing progressive and severe M-cone cell loss in the retina of −/− mice. (a′–f′) Magnifications of the superior/central inset regions of a–f. (a″–f″) 3D-reconstruction images from a′–f′. (**B–C**) Quantitative and temporal analyses of the number (**B**) and length of outer segments (**C**) of M-cone photoreceptors in the superior (S)/central regions of retina of −/− and +/− mice. (**D**) Comparison of topographic distribution of Cre^+^ cells in the retina of −/− and +/− mice at P13 showing the proximal localization of a few displaced cone cells in −/− mice. White dashed line represents a virtual midline boundary between proximal and distal areas of the outer nuclear layer (ONL) used for tallying the topography of Cre^+^-cell bodies in (**E**). (**E**) Quantitative and morphometric analyses of the localization of Cre^+^ cells in the proximal *versus* distal ONL and central *versus* peripheral retina between −/− and +/− mice. About 20% of Cre^+^ cells are present in the proximal ONL region of −/− at P13 and there is greater number of Cre^+^-cell loss in the central retina of −/− mice at P20. Legend: Boxes in **A** (a–f) are ROIs used for quantitation of data shown in **B** and **C**. Data shown represent the mean ± SD, *n* = 3–5; **, *p*<0.01; ***, *p*<0.001. Scale bars = 150 µm (a–f), 20 µm (a′–f′), 50 µm (**D**).

The degeneration of cone photoreceptors was quantitatively monitored in retinal flat mounts. We compared the number of M-cones and OS length between *HRGP-cre:Ranbp2^−/−^* and *HRGP-cre:Ranbp2^+/−^* mice (Figure 3). No differences were seen in the number of M-cones or in the length of their OS at P15. By P20, both measures were significantly decreased in *HRGP-cre:Ranbp2^−/−^* mice, and very few M-cones remained at P27 (Figure 3A–3C). Notably, the few surviving M-cones retained in P27 *HRGP-cre:Ranbp2^−/−^* mice still had OS which appeared of comparable length to those of *HRGP-cre:Ranbp2^+/−^* littermates (Figure 3A, 3C). At P20, the number of S-cones and the length of their OS were significantly decreased in *HRGP-cre:Ranbp2^−/−^* mice and prominent clumps of S-opsin were observed in the OS (Figure S3). We then examined the position of the Cre^+^-cell bodies within the proximal and distal ONL at peripheral and central regions of dorsal retinas (Figure 3D, 3E). Unlike *HRGP-cre:Ranbp2^+/−^* littermates, P13 *HRGP-cre:Ranbp2^−/−^* mice presented displaced Cre^+^-cell bodies in the peripheral and central regions of the proximal ONL. At P20, the number of Cre^+^-cell bodies had strongly decreased across the central and peripheral regions of the *HRGP-cre:Ranbp2^−/−^* retina, but this decrease was much more pronounced in the central retina (Figure 3E). By P27 and 3-months of age, respectively, very few and no Cre^+^ cells were observed in any regions of the retina (Figure 3E, data not shown).

### Non-autonomous death of rods upon demise of cone photoreceptors by distinct intrinsic mechanisms

**Figure 4:**
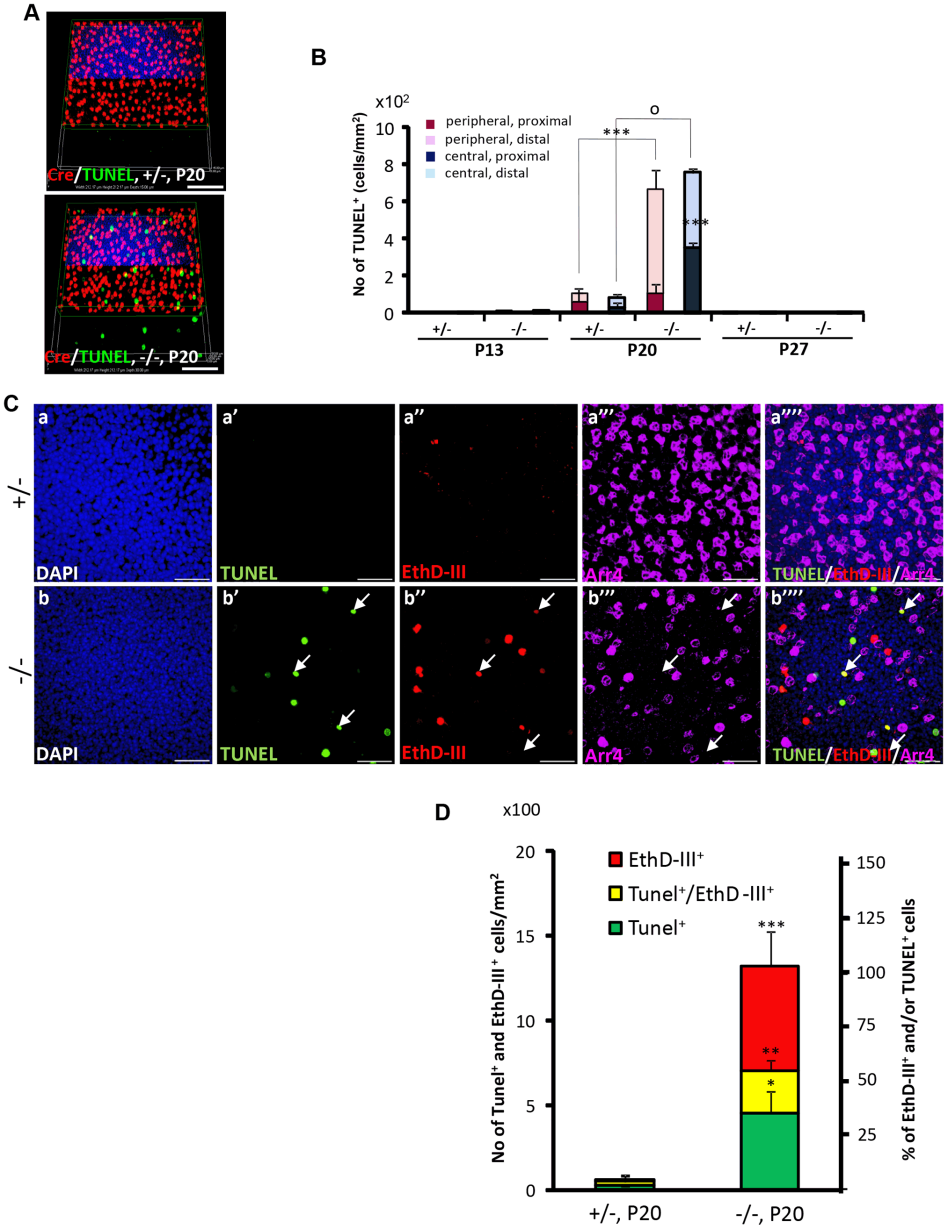
Spatiotemporal modalities of cell death between Cre^+^, Arr4^+^ and Arr4^−^-photoreceptor cell bodies in *HRGP-cre:Ranbp2^−/−^*. (**A**) Comparison of the relative 3-D distribution between Cre^+^-cone and TUNEL^+^ cells in the outer nuclear layer of retinas of *HRGP-cre:Ranbp2^+/−^* (+/−, top) and *HRGP-cre:Ranbp2^−/−^* mice (−/−, bottom) at P20 shows that all TUNEL^+^ cells are Cre^−^. (**B**) Morphometric and topographic distribution of TUNEL^+^ cells in outer nuclear layer (ONL, proximal *vs* distal) and regions of retina (peripheral *vs* central) between *HRGP-cre:Ranbp2^+/−^* (+/−) and *HRGP-cre:Ranbp2^−/−^* mice (−/−) at P13, P20 and P27. TUNEL^+^ cells become prominent at P20 with most localizing at the distal ONL of central and peripheral retina. No TUNEL^+^ cells were identified by P27. Data shown represent the mean ± SD, *n* = 4–5; ***, *p*<0.001; ^o^, *p*<0.0001. (**C**) Identification and localization of TUNEL^+^ (apoptotic) and EthD-III^+^ (necrotic) in Arr4^−^-photoreceptor cell bodies of P20 retinas of *HRGP-cre:Ranbp2^+/−^* mice (+/−; a–a′″) and *HRGP-cre:Ranbp2^−/−^* mice (−/−; b–b′″). Representative images of TUNEL^+^ (a′–b′) or EthDIII^+^ (a″–b″) cell bodies of Arr4^−^-photoreceptors (a′″–b′″) and co-localization of these (a″″–b″″) are shown. No TUNEL^+^ and EthD-III^+^-cell bodies in Arr4^+^-photoreceptor cell bodies were identified. Sections were counterstained with DAPI (a–b). White arrows in b′–b′″ indicate the localization of TUNEL^+^EthD-III^+^ cell bodies. (**D**) Quantification analysis of **C**. Among the Arr4^−^-photoreceptor cell bodies tallied, approximately 50, 30 and 20% were TUNEL^−^EthD-III^+^, TUNEL^+^EthD-III^−^ and TUNEL^+^EthD-III^+^, respectively, in −/− mice, whereas they were negligible in +/− mice. Data shown represent the mean ± SD, *n* = 3; ***, *p*<0.001; **, *p*<0.02; *, *p*<0.005; Scale bars = 50 µm (**A**), 20 µm (**C**).

OS shortening is thought to precede cell death of rod and cone photoreceptors [13], [28]. Hence, we employed multiple cell death markers to dissect out the molecular and subcellular processes underpinning the activation and progression of cell death between cone and rod photoreceptors of *HRGP-cre:Ranbp2^−/−^* mice. We used TUNEL staining to determine whether degenerating cone photoreceptors underwent apoptosis. As shown in Figures 4A and 4B, we did not identify any cell bodies that were TUNEL^+^ and Cre^+^. Instead, we found that all TUNEL^+^-cell bodies were Cre^−^, most likely rod photoreceptors, because these neurons comprise 97% of all photoreceptor cell types [59]. Morphometric analyses showed a drastic increase of TUNEL^+^-cell bodies at P20 (Figure 4B). Akin to the localization of Cre^+^ cells, this increase was significantly more pronounced also in central and peripheral regions of the distal ONL than in the counterpart proximal regions (Figure 4B).

To further differentiate TUNEL^+^-apoptotic cell bodies from necrotic cells among cone and rod photoreceptors, we carried out morphometric analysis of co-localization of TUNEL^+^ and cone Arr4^+^-cell bodies of retinal explants incubated with the membrane-impermeable and DNA-binding fluorescent dye, ethidium homodimer III (EthD-III). There were no Arr4^+^Tunel^+^ or Arr4^+^EthD-III^+^ cells in either genotype (Figure 4C). Instead, we found that *HRGP-cre:Ranbp2^−/−^* at P20 had about 70 and 55% of EthD-III^+^ Arr4^−^ and TUNEL^+^Arr4^−^ cells, respectively, with 30% of these cells being EthD-III^+^TUNEL^+^Arr4^−^ (Figure 4C, 4D). The presence of any of these apoptotic or necrotic cell bodies were negligible in *HRGP-cre:Ranp2^+/−^* (Figure 4C, 4D). These data support that the TUNEL^+^Arr4^−^, EthD-III^+^ Arr4^−^ and EthD-III^+^TUNEL^+^Arr4^−^ cells represent different cell death stages of rod photoreceptors.

**Figure 5:**
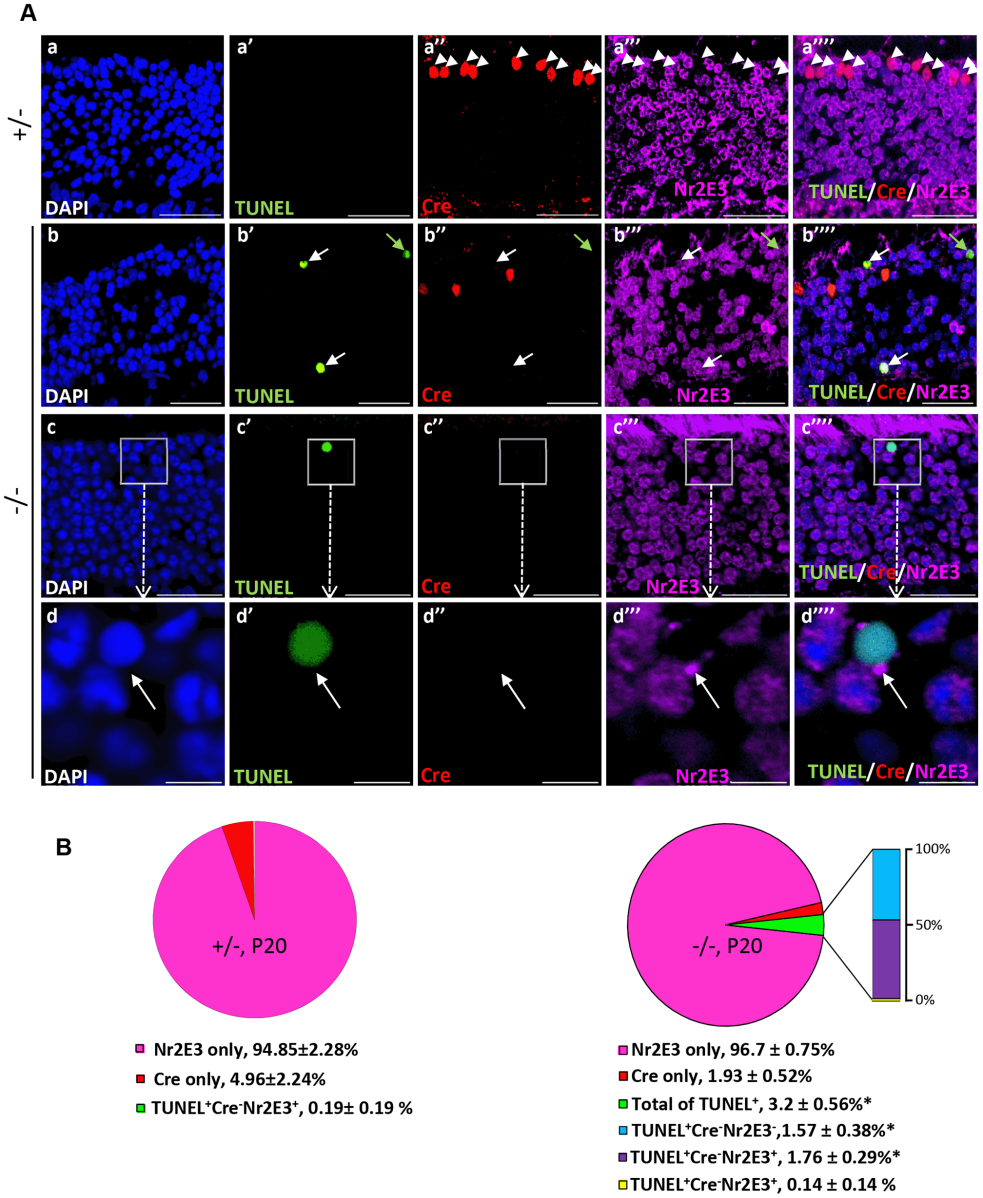
Cell death in Nr2E3^+^-cell bodies of rod photoreceptors in *HRGP-cre:Ranbp2^−/−^*. (**A**) Representative images of TUNEL^+^ (a′–d′), Cre^+^ (a″–d″) and Nr2E3^+^ (a′″–d′″) cell bodies and their colocalization (a″″–d″″) in rod photoreceptors of *HRGP-cre:Ranbp2^+/−^* (+/−; a–a″″) and *HRGP-cre:Ranbp2^−/−^* mice (−/−; b–d″″). d–d′″ are high magnifications of inset boxes of c–c″″. Sections were counterstained with DAPI (a–d). White arrowheads in a′–a″″ indicate Cre^+^Nr2E3^−^ cells, white and green arrows in b′–b″″ point to TUNEL^+^Nr2E3^+^ and TUNEL^+^Nr2E3^−^, respectively. (**B**) Quantification analysis of **A**. Among the TUNEL^+^-cell bodies tallied, slightly over 50% were Nr2E3^+^ in −/− mice, whereas they were negligible in +/− mice. No TUNEL^+^Cre^+^-cell bodies were identified in −/− mice. % values are based on % of total DAPI^+^ cells (100%). Data shown represent the mean ± SD, *n* = 3; *; *p*<0.001. Scale bars = 20 µm (a–c″″), 5 µm (d–d″″).

To establish unequivocally that TUNEL^+^Cre^−^-apoptotic cell bodies are rod photoreceptors neurons, retinal sections were co-immunostained for Nr2E3, a transcription factor specifically expressed in cell bodies of rod photoreceptors [60]. As shown in Figure 5A (a–b″″), all Cre^+^-cell bodies of either genotype were Nr2E3^−^, whereas many TUNEL^+^-cell bodies were Nr2E3^+^ in *HRGP-cre:Ranbp2^−/−^*. We also identified a small subpopulation of TUNEL^+^-cell bodies with Nr2E3 aggregation at discrete perinuclear foci (Figure 5A, c–d″″), suggesting that Nr2E3 localization changes and its expression decreases during rod photoreceptor death. Quantitative morphometric analysis of triple-immunostained retinas showed that about 3.2% of the total cells in *HRGP-cre:Ranbp2^−/−^* were TUNEL^+^, while 52% and 44% of these TUNEL^+^ cell bodies were Cre^−^NR2E3^+^ or Cre^−^Nr2E3^−^, respectively; the remaining 4% were dying rods with discrete perinuclear aggregation of Nr2E3 (Figure 5B). Further examination of the TUNEL^+^Nr2E3^−^ and TUNEL^+^Nr2E3^+^ cells indicated that they were TUNEL^+^EthD-III^+^ or TUNEL^+^EthD-III^−^ (Figure S4). These data support that TUNEL^+^Nr2E3^+^ and TUNEL^+^Nr2E3^−^ cells also represent different stages of cell death of rod photoreceptors.

**Figure 6:**
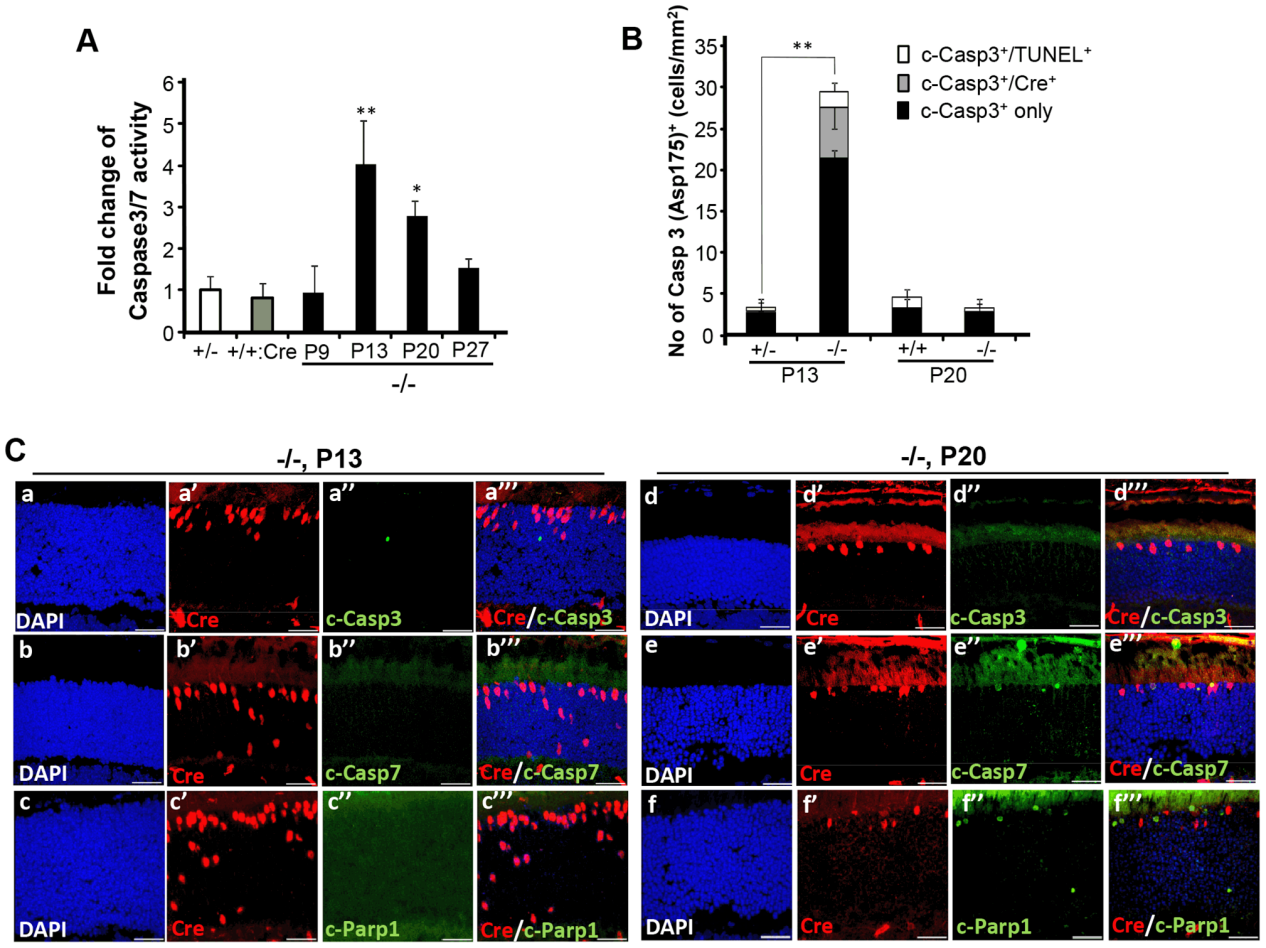
Spatiotemporal activation of caspases 3 and 7 and Parp1 in photoreceptors of *HRGP-cre:Ranbp2^−/−^* mice. (**A**) Temporal profile of caspase 3/7 activation of retinal extracts of *HRGP-cre:Ranbp2^−/−^* mice (−/−) mice relative to age-matched +/− controls shows significant caspase3/7 activation at P13 and P20. The caspase 3/7 activities of +/− and +/+:Cre controls shown are at P20. (**B**) Quantitative, temporal and relative distributions between activated (cleaved) caspase 3 (c-casp3^+^), Cre^+^ and TUNEL^+^ in cell bodies of photoreceptors of *HRGP-cre:Ranbp2^+/−^* (+/−) and *HRGP-cre:Ranbp2^−/−^* mice (−/−) at P13 and P20. The majority of photoreceptor cell bodies are c-casp3^+^Cre^−^. The levels of any type of c-casp3^+^ cell bodies are negligible by P20. (**C**) Relative immunolocalizations of Cre^+^ and c-casp3^+^ (a–a′″, d–d′″), Cre^+^ and c-casp7^+^ (b–b′″, e–e′″), and Cre^+^ and activated Parp1 (c-Parp1^+^) (c–c′″, f–f′″) cell bodies of photoreceptors of *HRGP-cre:Ranbp2^−/−^* mice at P13 (a–c′″) and P20 (d–f′″). c-casp3^+^ develop at P13, c-casp7^+^ and c-Parp1^+^ at P20 and all c-Parp1^+^ cell bodies are Cre^−^, whereas c-casp7^+^ are Cre^+^. Legend: Data shown represent the mean ± SD, *n* = 4–5; *; *p*<0.05, **; *p*<0.01; scale bars = 25 µm; cleaved caspases 3 and 7, c-casp3 and c-casp7, respectively.

We next examined whether cone and rod photoreceptor degenerations were accompanied by the activation of caspases, a cardinal feature of cell death [61], [62]. We screened whole retinal extracts of *HRGP-cre:Ranbp2^−/−^* mice at P20 with substrates against specific caspases and found strong activation of caspases 3/7, mild activation of caspases 8 and 9, and no activation of caspases 1, 2 and 6, when compared to *HRGP-cre:Ranbp2^+/−^* mice (Figure S5). To define the spatiotemporal profile of caspase activation, we examined the activities of caspases 3 and 7 in retinal extracts and carried out morphometric analyses of retinal sections immunostained with antibodies against cleaved (activated) caspase 3, 7 or 9 of age-matched mice of both genotypes. In *HRGP-cre:Ranbp2^−/−^* mice, we found that the activities of caspase 3, caspase 7, or both, peaked at P13 (Figure 6A), well before the rise in TUNEL^+^-cell bodies in the ONL (Figure 4B), and that these activities were negligible by P27, when most cones had died (Figure 6A). To distinguish caspase 3 and 7 activities, we immunostained retinal sections of different ages for active caspase 3 and 7 and performed morphometric analysis (Figure 6B, 6C). These experiments showed that the majority of active caspase 3^+^-photoreceptor cell bodies were Cre^−^TUNEL^−^ (Figure 6B), but there was also a small but significant fraction of active caspase-3^+^Cre^+^-cell bodies at P13 in *HRGP-cre:Ranbp2^−/−^* mice (Figure 6B, 6C). All types of active caspase 3^+^-photoreceptor cell bodies were drastically decreased in *HRGP-cre:Ranbp2^−/−^* mice and their presence were no different from *HRGP-cre:Ranbp2^+/−^* by P20 (Figure 6B, 6C a–a′″, d–d′″). By contrast, caspase 7^+^-cell bodies became prominent only at P20 and most were Cre^+^ (Figure 6C, b′–b′″, e′–e′″). Further, the temporal profiles of caspase 3 and 7 activities paralleled their transcriptional up-regulation (Figure S6). Collectively, the data support that activation of caspase 3 in rods and caspase 7 in cones contribute to the rise of activities of caspases 3 and 7 at P13 and P20, respectively.

Next, we examined the activation of Parp1 (Parp1^+^; Figure 6C, Figure S7), which is cleaved during apoptosis into 89 and 24 kDa fragments by caspase 3 or 7 [63], [64] and has been linked to rod photoreceptor degeneration [65]. We found that Parp1^+^cells were never Cre^+^ and that their appearance was biphasic with activity peaks at P9 and P20 (Figure 6C, Figure S7A, a–b″″). These temporal peaks of Parp1^+^-activities in rod photoreceptors coincided with the activation of caspase 9 in rods at P9 (Figure S7A, c–d″″) and caspase 7 in cones at P20 (Figure 6C). No Parp1^+^-cell bodies could be identified at P13 (Figure 6C). At P9, caspase 9^+^TUNEL^+^ and Parp1^+^TUNEL^+^ cell bodies were always cre^−^ rods, while TUNEL^+^caspase 9^−^cre^−^ and TUNEL^+^Parp1^−^cre^−^ rods could also be observed (Figure S7A). These events indicate that caspase 9 and Parp1 activation in rod photoreceptors is short-lived. These manifestations were accompanied by transcriptional up-regulation of caspase 9 and Parp1 between P9 and P20, but not of caspase 8, which is typically activated by extrinsic cell-death mechanisms (Figure S7B) [61], [62].

The formation of rod apoptotic cell bodies prompted us to examine whether apoptosis-inducing factor (AIF) or cytochrome *c* was released from the mitochondria, as these are also hallmark events of the apoptotic cascade [66]–[68]. Subcellular fractionation of retinas showed no sign of AIF or cytochrome *c* in the cytosol fraction of either genotype at the peak of apoptosis (Figure S8A). Changes in AIF levels were also not observed in the nuclear-enriched fraction of either genotype (Figure S8B). In addition, the expression levels of the apoptotic protease activating factor-1 (Apaf-1) remained unchanged (data not shown). Finally, we examined markers for necroptosis, such as members of the receptor-interacting proteins, RIP1 and RIP3, and macroautophagy, such as the autophagosomal membrane marker, light chain 3B II (LC3B II), since they are thought to be induced upon photoreceptor degeneration [69]–[71]. Immunoassays of retinal extracts found no differences in these markers between *HRGP-cre:Ranbp^+/−^* and *HRGP-cre:Ranbp2^−/−^* mice at P13 (Figure S8C, S8D).

### Selective activation of MMP11 in *HRGP-cre:Ranbp2^−/−^* mice

**Figure 7:**
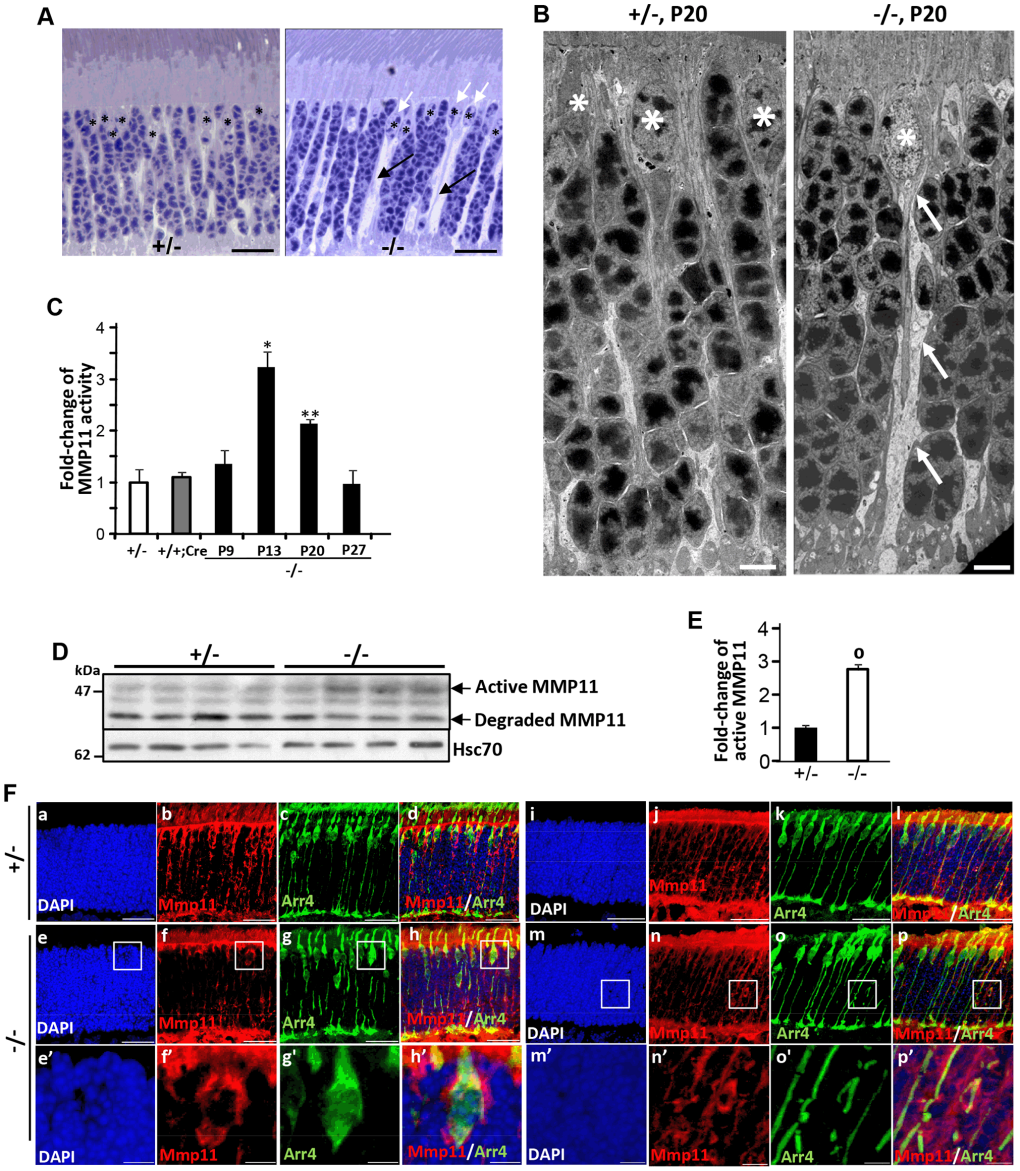
Activation of matrix metalloproteinase 11 (MMP11) upon ablation of *Ranbp2* in cone photoreceptors. (**A**) Light microscopy images of methylene blue stained semi-thin retinal sections showing the presence of prominent interstitial spaces (black arrows) originating from cone cell bodies (white arrows) and between cell bodies of rod photoreceptors in *HRGP-cre:Ranbp2^−/−^* (−/−) compared to *HRGP-cre:Ranbp2^+/−^* (+/−) mice. * denote euchromatic nuclei of cones. (**B**) Electron micrographs depicting ultrastructural changes in the lower fiber of a −/− cone photoreceptor. Note the swelling of the lower fiber (arrows) traced to the cell body of a cone nucleus characterized by its prominent euchromatin (*). *, cone nuclei. (**C**) Temporal profile of MMP11 activity of retinal extracts of *HRGP-cre:Ranbp2^−/−^* mice (−/−) mice relative to age-matched +/− controls shows significant MMP11 activity at P13 and P20. The MMP11 activity of +/− and +/+:Cre controls shown are at P20. (**D**) Immunoblots of MMP11 from retinal homogenates of *HRGP-cre:Ranbp2^−/−^* (−/−) compared to +/− mice at P20 showing an increase of the active form of MMP11 in −/−. Hsc70 is cytosolic heat shock protein 70 used as loading control. (**E**) Quantitation analysis of the levels of active MMP11 in (**D**) of *HRGP-cre:Ranbp2^−/−^* (−/−) relative to age-matched +/− mice at P20. (**F**) Retinal sections immunostained with antibodies against cone arrestin (Arr4) and MMP11. (a–d) central retinal region of *HRGP-cre:Ranbp2^+/−^* (+/−); (e–f) central retinal region of *HRGP-cre:Ranbp2^−/−^* (−/−); (e′–f′) magnification of boxed regions shown in e–f; (i**–**l) peripheral retinal region of *HRGP-cre:Ranbp2^+/−^* (+/−); (m**–**p) peripheral retinal region of *HRGP-cre:Ranbp2^−/−^* (−/−); (m′–p′) magnification of lower fibers of cones of boxed regions shown in m–p. MMP11 predominantly localizes to cone photoreceptors, such as interstitial space around cell bodies, lower fibers and inner segments. Legend: Data shown represent the mean ± SD, *n* = 4; *, *p*<0.05; **, *p*<0.01; ^o^, *p*<0.0001; scale bars = 25 µm (A, F), 5 µm (B).

Histological examination of semi-thin retinal sections showed that retinas of *HRGP-cre:Ranbp2^−/−^* developed prominent interstitial spaces between photoreceptors cell bodies and across the ONL by P20, a phenotype not present in *HRGP-cre:Ranbp2^+/−^* mice (Figure 7A). The interstitial spaces could always be traced to prominent euchromatic nuclei typically localized at the distal (outer) edge of the ONL (Figure 7A), hallmark morphological and topographic features of cell bodies of cone photoreceptors. Detailed examination of the ultrastructure of the ONL showed that the interstitial spaces reflect degenerating lower fibers of cone photoreceptors that were dilated and very lucent (Figure 7B). This striking phenotype led us to hypothesize that ablation of *Ranbp2* in cones promotes the activation of metalloproteinase(s) (MMPs) causing the weakening and degradation of the extracellular matrix, which normally organizes photoreceptor cell bodies within the ONL [72], and may contribute to the retraction of Cre^+^-cell bodies from the distal (outer) to the proximal (inner) region of the ONL (Figure 2). Hence, we screened retinal extracts for each the eleven MMP activities. In comparison to *HRGP-cre:Ranbp2^+/−^*, retinal extracts of *HRGP-cre:Ranbp2^−/−^* mice presented ∼3-fold higher activity of MMP11, but not of any other MMPs (Figure S9A). MMP11 activity was elevated at P13 and P20 and returned to control levels at P27 when most cones have degenerated (Figure 7C). The increase of MMP11 activity was also accompanied by a ∼3-fold increase of the active form of MMP11 (Figure 7D, 7E). The transcriptional up-regulation of *Mmp11* in *HRGP-cre:Ranbp2^−/−^* mice as early as P9 followed its transient down-regulation at P7 and preceded the activation of MMP11 at P13, whereas the transcriptional levels of *Timp3* remained largely unchanged across different ages (Figure S9B, 7C). To assess the cellular origin of MMP11 expression and activity, we performed immunohistochemistry of MMP-11 in retinal sections from P20 mice, and found that MMP-11 was localized prominently around cell bodies, inner segments and lower fibers of cone Arr4^+^-cells (Figure 7F). Collectively, these data confirm that ablation of *Ranbp2* in cones promotes the up-regulation of MMP11 expression and activity in these neurons, an event which likely contributes to the development of interstitial spaces in the ONL and retraction of cones cell bodies to the proximal ONL in *HRGP-cre:Ranbp2^−/−^* mice.

### Erosive ultrastructural destruction of cones and damage of rod photoreceptors

**Figure 8:**
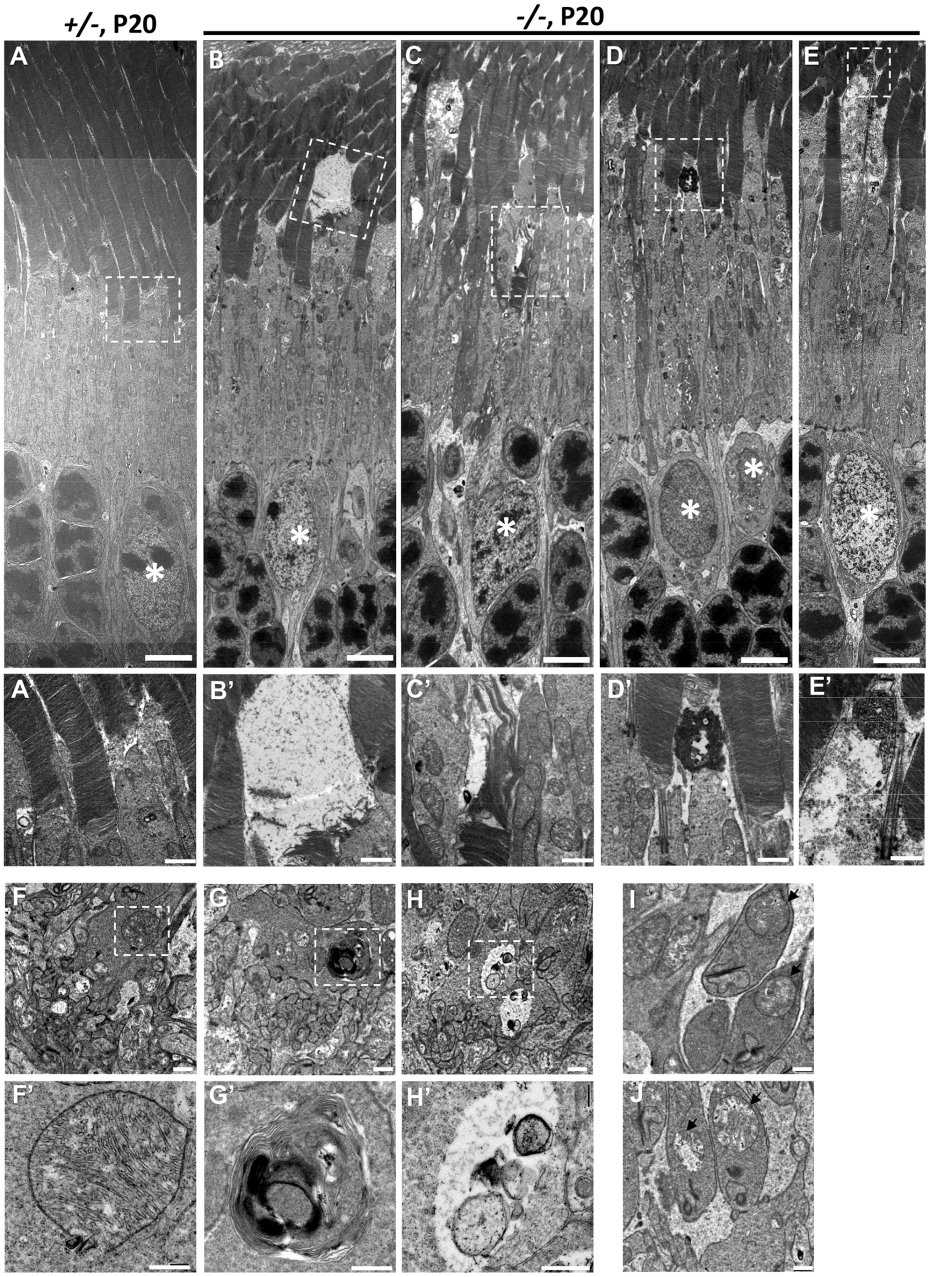
Ultrastructural changes of cone and rod photoreceptors upon ablation of *Ranbp2* in cones at P20. (**A**) Ultrastructural image of rod and cone photoreceptors outer and inner segments of *HRGP-cre:Ranbp2^+/−^* (+/−). All structures look unremarkable. (**B–E**) Representative ultrastructural images of multiple features rod and cone photoreceptors outer and inner segments of *HRGP-cre:Ranbp2^−/−^* (−/−). (**B**) shows the collapse of the cone outer segment and formation of a large electron lucent area, (**C**) shows extended discs and partial erosion of cone outer segment, (**D**) shows the accumulation of amorphous electron dense material at the connecting cilium of a cone photoreceptor, (**E**) shows the formation of electron lucent areas in the inner segment of a cone photoreceptor. White stars, euchromatic nuclei of cone photoreceptors. (**A′–E′**) are magnifications of boxed areas in **A–E** showing the aforementioned pathological features. (**F**) Synaptic pedicles of cones with normal morphology of *HRGP-cre:Ranbp2^+/−^* (+/−). (**G**) Multi-lamellar body with electrodense material in a cone synaptic pedicle of *HRGP-cre:Ranbp2^−/−^* (−/−). (**H**) Electron lucent area surrounding subcellular debris in a cone synaptic pedicle of *HRGP-cre:Ranbp2^−/−^* (−/−). **F′–H′** are magnified images of boxed areas in **F–H**. (**I**) Synaptic spherule of a rod photoreceptor containing unremarkable mitochondria (black arrows) in *HRGP-cre:Ranbp2^+/−^* (+/−). (**J**) Synaptic spherule of a rod photoreceptor with abnormal mitochondria (black arrows) containing disorganized cristae and electron lucent areas in the matrix in *HRGP-cre:Ranbp2^−/−^* (−/−). Scale bars =  5 µm (**A–E**), 1 µm (**A′–E′**), 500 nm (**F–H**) and 200 nm (**F′–H′**).

To ascertain in greater detail the morphological changes of degenerating cone photoreceptors, we examined the ultrastructure of *HRGP-cre:Ranbp2^+/−^* and *HRGP-cre:Ranbp2^−/−^* retinal sections at P20. The subcellular subcompartments of cone and rod photoreceptors of *HRGP-cre:Ranbp2^+/−^* mice had normal morphologies (Figure 8A, 8A′, 8F, 8F′, 8I), whereas cone photoreceptors of *HRGP-cre:Ranbp2^−/−^* mice exhibited distinct morphological changes across multiple subcellular compartments. Across different photoreceptor cells of *HRGP-cre:Ranbp2^−/−^*, we found that cone OS membranes were extended, disorganized and collapsed into large, amorphous and lucent areas (Figure 8B, 8B′, 8C, 8C′), that electrodense material accumulated at the connecting cilium (Figure 8D, 8D′), and that prominent electron lucent areas were present in the inner segments (Figure 8E, 8E′). Additional abnormalities were seen at the synaptic pedicles including accumulation of multilamellar bodies (Figure 8G, 8G′), mitochondria with widespread electron lucent matrix areas without cristae and formation of cytosolic lucent areas without a limiting membrane around subcellular debris (Figure 8H, 8H′). Albeit less extensive, cristae of some mitochondria in the rod synaptic spherules were also disrupted resulting in the formation of lucent areas within the matrix (Figure 8J).

### Multiphasic and temporal changes in gene expression and pathways upon *Ranbp2* ablation

**Figure 9:**
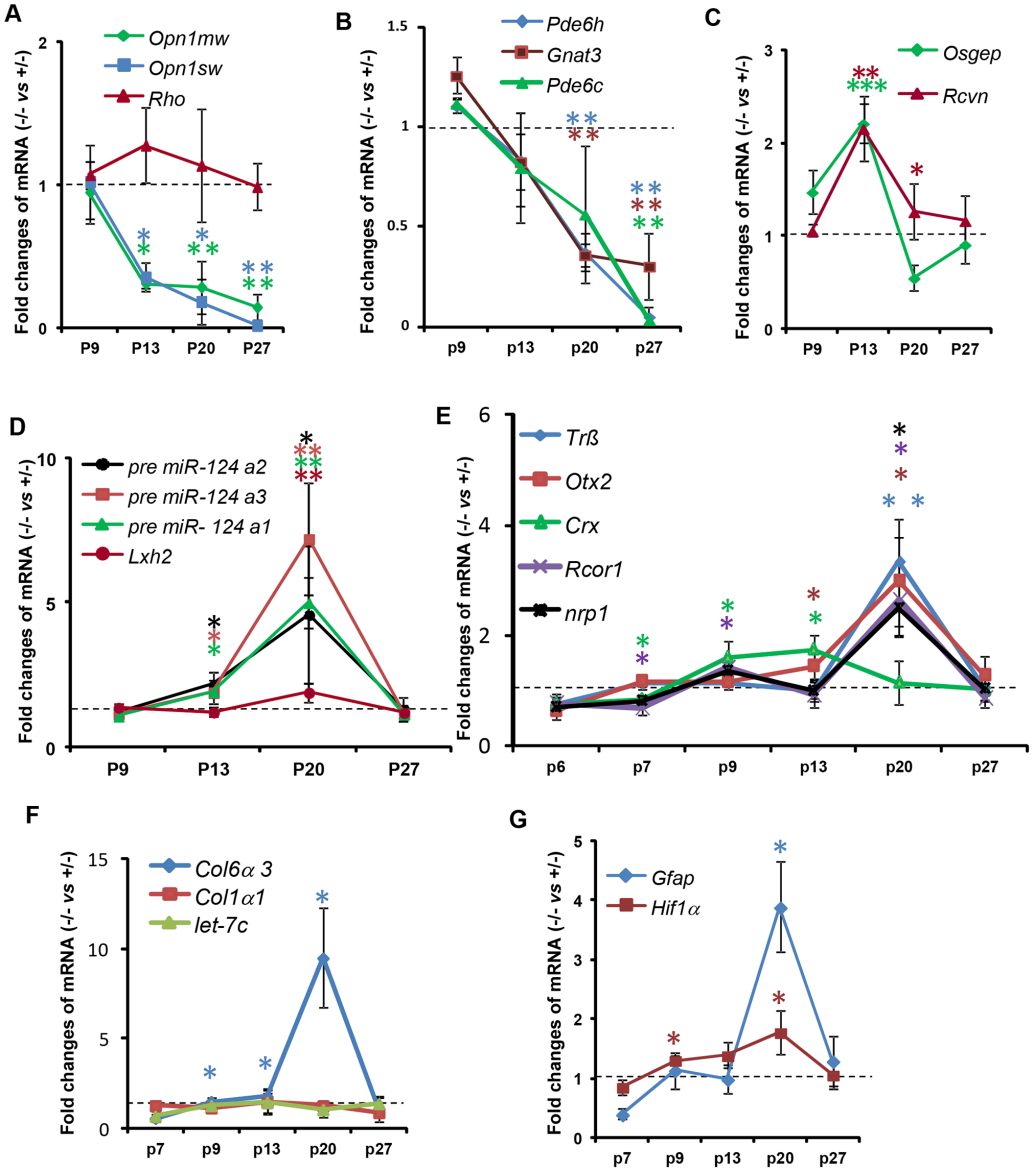
Temporal profiles of changes in gene expression by ablation of *Ranbp2* in cone photoreceptors. (**A–G**) qRT-PCR of cone-specific (**A, B**), pan-photoreceptor (**C**), cone survival (**D**) and cone transcription factors (**E**), *col6α3* and *col1α1* (**F**), and *Gfap* and *Hif1α* transcripts (**G**). There is a decrease of cone-specific transcripts beginning at P13 (**A, B**), whereas pan-photoreceptor (**C**) and cone survival genes (**D**) begin transient up-regulations at the same age. The expression of cone survival genes peaks at P20 (**D**). (**E**) The transcription factors, *Crx* and *CoREST* (*Rcor1*), are the first nuclear factors whose changes of their transcriptional levels coincides with the genetic ablation of *Ranbp2* at P7. These events are followed by the up-regulations at P20 of the transcription factors, *Nrp1*, *Trß2* and *Otx2* (**E**). The up-regulations of transcripts encoding the substrate of MMP11, *col6α3*, begins at P9 and peaks at P20 (**F**), whereas those for the hypoxia marker, *Hif1α*, and inflammatory marker, *Gfap*, begin at P9 and P20, respectively (**G**). Legends: Data shown represent the mean ± SD, *n = *3–4; *, *p*<0.05; **, *p*<0.01; ***, *p*<0.001; refer to table S1 for gene designations/symbols.

We examined the effect of *Ranbp2* ablation selectively in cones on the expression of cone photoreceptor-specific and other pertinent genes by quantitative real time-PCR (qRT-PCR) (Figure 9, Table S1). We found that *Ranbp2* ablation led to rapid declines of M-opsin (*Opn1m*w) and S-opsin (*Opn1s*w) mRNAs as early as P13 (Figure 9A), when there are yet no prominent cellular changes in M-opsin and Cre^+^-neurons between genotypes (Figure 3B, 3E), and such declines occurred without concomitant changes in rhodopsin (*Rho*) mRNA (Figure 9A). Other cone-specific genes showed a similar pattern of down-regulation, including *Pde6h*, *Pde6c* and *Gnat3* (Figure 9B). By P27, when cones have degenerated, the expression of all cone-specific genes was at or below the detection limit in *HRGP-cre:Ranbp2^−/−^* retinas (Figure 9A, 9B). By contrast, the expression of the pan-photoreceptor markers, *Rcvn* and *Osgep*, increased transiently at P13 (Figure 9C).

We also examined the genetic variants of *miR-124a* (*miR-124a1/Rncr3*, *miR-124a2*, *miR-124a3*), which mediate cone survival, and its downstream target transcript, *Lhx2*, whose translation is suppressed by *miR-124a* [73]. We found strong up-regulation of *miR-124a* in the *HRGP-cre:Ranbp2^−/−^* retina, which was accompanied by increased levels of *Lhx2*, albeit of a lesser magnitude (Figure 9D). The time course of these changes was similar, beginning at P13, peaking at P20 and returning to *HRGP-cre:Ranbp2^+/−^* levels at P27 when cones have degenerated (Figure 9D). We also assessed the expression of *Trß2*, *Otx2* and *Crx*, encoding transcription factors critical to the maturation and maintenance of cone photoreceptors (Figure 9E) [74]–[78]. *Crx* expression was decreased at P7, when Cre-mediated excision of *Ranbp2* occurs (Figure 1C), and then rose steadily up to P13, and then decreased to *HRGP-cre:Ranbp2^+/−^* levels at P27. In comparison, expression of *Trß2* and *Otx2* increased later, with a sharp peak at P20, and then declined to basal levels at P27 (Figure 9E). Like *Crx*, *CoREST* (also known as *Rcor1*), a cofactor of REST (repressor element 1 silencing transcription factor), was transiently down-regulated at P7 and then rose at P9 until P20, when there was a transient up-regulation of neuropilin-1 (*nrp1*), a receptor whose transcriptional expression is modulated by miR-124 and CoREST (Figure 9E) [79]. The expressions of *CoREST* and *nrp1* also returned to basal levels at P27 (Figure 9E).

Because *Ranbp2* ablation induced the expression and activation of MMP11, a metalloproteinase which is known to cleave selectively the α3 chain of collagen VI (*Col6α3*) [80], we examined the time course changes of *Col6α3* expression in the *HRGP-cre:Ranbp2^−/−^* retina. A selective rise in *Col6α3* expression, but not α1 chain of collagen I (*Col1α1*), was detected as early as P9, shortly after a transcriptional increase in *Mmp11* was also detected (Figure 9F, S9B). *Cola6α3* expression peaked at P20 and then became indistinguishable from *HRGP-cre:Ranbp2^+/−^* mice at P27. We did not observe any transcriptional changes in *let-7c*, a miRNA reported to promote the down-regulation of *Mmp11* (Figure 9F) [81]. Finally, we examined transcriptional changes in markers associated with neurodegenerative mechanisms including with autophagy (e.g. cathepsin S, lysozyme and culsterin), glycolysis (*6-Pfk*), hypoxia (*Hif1α*) and inflammation (*Gfap*). Among these, we found changes only in *Hif1α* and *Gfap* with *Hif1α* rising from P9 until P20 and *Gfap* transiently spiking at P20 when most cones are undergoing degeneration (Figure 9G).

**Figure 10:**
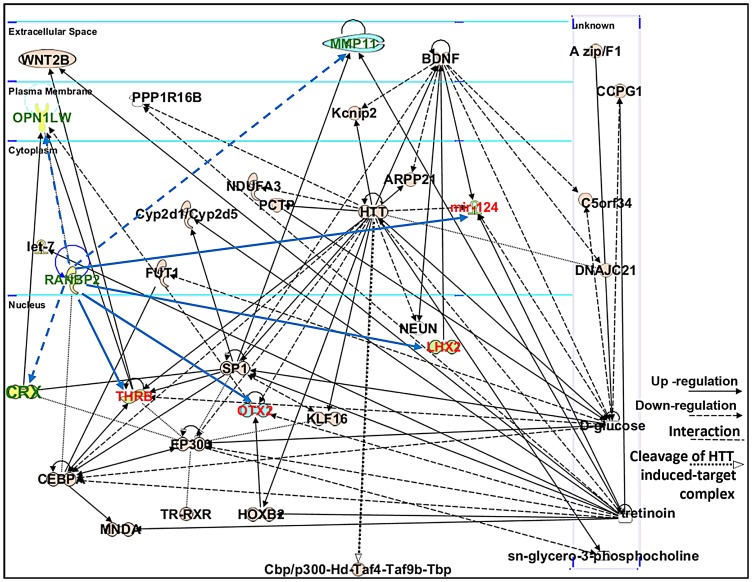
Connectivity map of a gene network linked to Ranbp2 functions. Ingenuity pathway analysis (IPA) of factors modulated by loss of Ranbp2 identified a top network implicated in “Cellular and Nervous System Development and Function, and Carbohydrate Metabolism” (score of 22). Dashed and solid blue arrows were added manually to the network to reflect the down-regulation and up-regulation of genes (shown in green and red), respectively, observed first upon ablation of *Ranbp2* in cone photoreceptors as identified by this work. Note that *Crx* and *Mpp11* expressions underwent first transient down-modulation at P7 followed by their sustained up-regulation. Legend: ARPP21, cAMP-regulated phosphoprotein, 21 kDa; BDNF, Brain derived neurotrophic factor; C5orf34, chromosome 5 open reading frame 34; cbp, CREB-binding protein; CCPG1, cell cycle progression 1; CEBPA, CCAAT/enhancer-binding protein alpha; CRX, Cone-rod homeobox protein; Cyp2d1/Cyp2d5, cytochrome P450, family 2, subfamily d, polypeptide 1/cytochrome P450, family 2, subfamily d, polypeptide 5; DNAJC21, DnaJ homolog subfamily C member 21; EP300, also called p300, E1A binding protein p300; FUT1, fucosyltransferase 1; HOXB2, homeobox B2; HTT or Hd, huntingtin; Kcnip2, K_v_ channel-interacting protein 2; KLF16, Kruppel-like factor 16; let-7, Let-7 microRNA precursor; LHX2, LIM/homeobox protein2; mir-124, microRNA 124; MMP11, Matrix Metalloproteinase 11; MNDA, myeloid cell nuclear differentiation antigen; NDUFA3, NADH dehydrogenase (ubiquinone) 1 alpha subcomplex, 3; NEUN, Feminizing Locus on X-3, Fox-3, or Hexaribonucleotide Binding Protein-3; OPN1LW, long-wave-sensitive opsin (cone pigment); OTX2, orthodenticle homeobox 2; PCTP, Phosphatidylcholine transfer protein; PPP1R16B, protein phosphatase 1, regulatory subunit 16B; sn-glycero-3-phosphocholine, 2,3-dihydroxypropyl 2-(trimethylazaniumyl)ethyl phosphate; SP1, Sp1 transcription factor; THRB, thyroid hormone receptor, beta; Taf4, TAF4 RNA polymerase II, TATA box binding protein (TBP)-associated factor; Taf9b, TAF9B RNA polymerase II, TATA box binding protein (TBP)-associated factor; Tbp, TATA box binding protein; TR, Thyroid Hormone Receptor; RXR, Retinoic acid receptor; tretinoin, all-*trans* retinoic acid or ATRA; WNT2B, wingless-type MMTV integration site (WNT) family2B.

Ranbp2 has multifaceted roles in biology and pathology across tissues, cell types and cell-stages. This complexity reflects the interaction of the diverse structural modules of Ranbp2 with multifunctional partners. Hence, we employed the Ingenuity pathway analysis (IPA) to identify and delineate connectivity maps linking Ranbp2 with the regulation of expression of genes/proteins identified by this study and genetic, protein and metabolic networks. The top network hit generated by IPA (score 22) was associated to “Cellular Development, Nervous System Development and Function and Carbohydrate Metabolism” (Figure 10). This network comprised thirty-two gene products and three endogenous chemicals, D-glucose, *sn*-glycero-3-phosphocholine and tretinoin (all-*trans* retinoic acid) (Figure 10). Remarkably, this connectivity map revealed links between transcription factors, many of which belong to the orphan nuclear receptor family and are known to be modulated by Ranbp2 levels, and metabolites, whose levels are also affected by Ranbp2 and regulate transcriptional activities of nuclear factors [47], [49], [51], [82], [83]. Among other novel points of interest, the IPA revealed central roles of *i)* huntingtin cross-talk with nuclear factors modulated by Ranbp2, a mechanism which is thought to be disrupted in Huntington's disease (HD) [84], and *ii)* two secreted and extracellular signaling proteins, wingless-type MMTV integration site family, member 2 (WNT2B) and brain-derived neurotrophic factor (BDNF), intersecting multiple nodes modulated by Ranbp2 and other factors. WNT2B and BDNF are known to play important roles in regulation of cell growth, differentiation, tumorigenesis, and to support the survival of existing neurons, respectively [85]–[87].

### Retinal physiological deficits upon impairment and loss of cone photoreceptors

**Figure 11:**
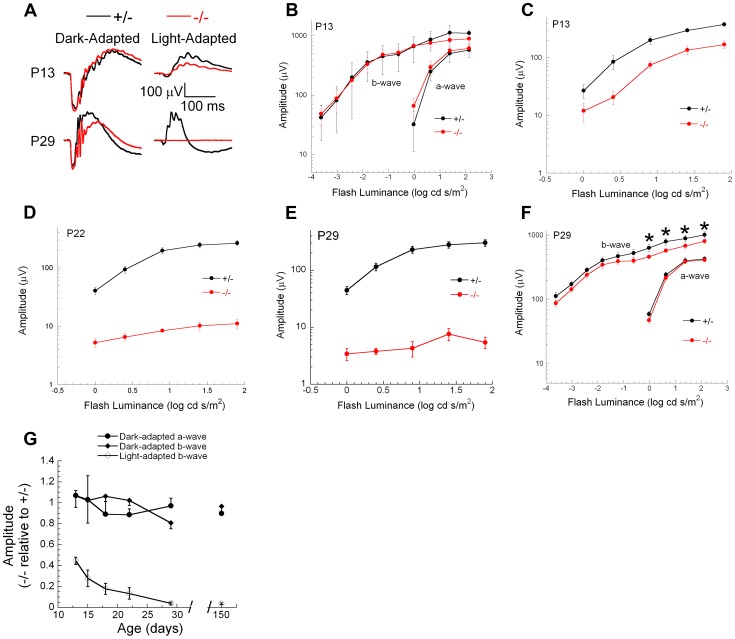
Electrophysiological responses of cones and rod photoreceptors upon ablation of *Ranbp2* in cones. (**A**) Representative electroretinograms (ERGs) of control *HRGP-cre:Ranbp2^+/−^* (+/−, black) and *HRGP-cre:Ranbp2^−/−^* mice (−/−, red) mice at P13 and P29 to a 1.4 log cd s/m^2^ stimulus flash presented in darkness (left) or superimposed upon a steady rod-desensitizing adapting field (right). (**B–F**) Luminance-response functions for the major components of the dark-adapted rod (**B, F**) or light-adapted cone ERGs (**C–E**) at P13 (**B, C**), P22 (**D**) or P29 (**E, F**). Repeated measures analysis of variance was used to compare luminance-response functions of +/− and −/− mice for the amplitude of the major ERG components. Cone ERG amplitudes were significantly reduced in −/− mice at all ages examined (P13, P18, P22, P29, P150). Dark-adapted *a*-wave amplitudes were not different between +/− and −/− mice at any age examined. Dark-adapted *b*-wave luminance-response functions were also not different between +/− and −/− mice at any age examined. At P29 (**F**) and P150, dark-adapted ERG *b*-waves were significantly reduced (*p*<0.05, t-test, denoted by *) for stimuli above 0.0 log cd s/m^2^, but not for lower luminance stimuli. (**G**) Amplitude of −/− responses expressed relative to those of +/− littermates for the major ERG response components. Each measure reflects a single stimulus condition: ‘Light-adapted *b*-wave’ represents cone ERGs obtained to a 1.4 log cd s/m^2^ stimulus flash superimposed upon a steady adapting field; ‘Dark-adapted *a*-wave’ represents rod ERG *a*-waves obtained to a 1.4 log cd s/m^2^ stimulus flash presented in darkness; ‘Dark-adapted b-wave’ represents rod ERG *b*-waves obtained to a −1.2 log cd s/m^2^ stimulus flash presented in darkness. Data points indicate the average ± s.e.m. for *n* = 5–10 mice.

Genetic excision of *Ranbp2* is detectable by P7, which coincides with immediate transcriptional changes in the levels of *Mmp11*, *Crx* and *CoREST* (Figure 9E, S9B). Reduced levels of cone-specific transcripts, such as those encoding phototransduction proteins, were not detectable until P13 and changes in cone morphology were not observed until P20. These observations, and the non-autonomous molecular and cellular effects of cones on rod photoreceptors, prompted us to determine the onset and progression of cone and rod physiological dysfunction caused by ablation of *Ranbp2* in cones. We measured cone and rod function by light- and dark-adapted electroretinograms (ERGs), respectively. Figure 11 summarizes the ERG data obtained from mice between P13 and 150 days. At P13, dark-adapted ERGs of *HRGP-cre:Ranbp^+/−^* and *HRGP-cre:Ranbp2^−/−^* mice were comparable (Figure 11A, left; Figure 11B), indicating equivalent retention of rod-mediated outer retinal activity at this age. In comparison, light-adapted ERGs, reflecting cone activity [88], [89] were already reduced significantly at P13 (Figure 11A, right; Figure 11C). By P22, the amplitude of the light-adapted ERGs were markedly reduced in *HRGP-cre:Ranbp2^−/−^* mice (Figures 11D), and cone ERGs were extinguished in *HRGP-cre:Ranbp2^−/−^* mice at P29 (Figure 11A, 11E). The amplitude of the dark-adapted ERG *a*-wave, which reflects phototransduction in the outer segments of rod photoreceptors, was not significantly different between *HRGP-cre:Ranbp2^+/−^* and *HRGP-cre:Ranbp2^−/−^* mice at any age examined (Figure 11A, 11B, 11F, 11G). In mice aged P29 and P150, the dark-adapted ERG *b*-wave was reduced, but in a luminance-dependent fashion (Figure 11F, 11G). At low stimulus luminances, where the *b*-wave reflects synaptic transmission from rod photoreceptors to rod bipolar cells, there was no significant reduction in amplitude. At higher stimulus luminances, where both rod- and cone-mediated synaptic activity contribute to the *b*-wave, a significant amplitude reduction was observed (Figure 11F). The synaptic localization of post-synaptic density 95 (PSD95) protein was comparable between genotypes at P20 (Figure S10), indicating that the *b*-wave reductions noted at high flash luminances reflects a loss of the cone pathway contribution to the ERG *b*-wave at these stimulus levels, and not to an alteration in synaptic transmission between rod photoreceptors and bipolar cells. Figure 11G plots the amplitude of *HRGP-cre:Ranbp2^−/−^* light- and dark-adapted ERG components relative to those of control littermates (*HRGP-cre:Ranbp2^+/−^*) ranging from P13 to 21-weeks in age. *HRGP-cre:Ranbp2^−/−^* mice have a selective reduction in the light-adapted ERG indicating that the dark-adapted ERG is not sensitive to the limited cone-induced rod loss seen by other cell biological measures previously described in this study. In agreement with the electrophysiological observations, the complete loss of cone photoreceptors and the limited cone-induced loss of rod photoreceptors did not cause significant changes in ONL thickness and cell body density when analyzed by light microscopy in mice as old as 12-weeks of age (Figure S11).

## Discussion

The results of this study demonstrate: *i)* cone-specific ablation of *Ranbp2* triggers the demise of cone photoreceptors with concomitant non-autonomous cell death of rod photoreceptors, *ii)* the death of rod photoreceptors is contingent on the presence of cone photoreceptors, and *iii)* the cell death mechanisms of cone and rod photoreceptors are intrinsically distinct with the former and latter undergoing atypical features of necrosis and apoptosis, respectively [61], [62]. We found that cone photoreceptors undergo necrotic changes including massive erosive destruction of their intracellular contents and late caspase 7 activation, but without apparent changes in loss of membrane permeability. In comparison, rods undergo apoptosis, caspase 3 and Parp1 activations, and loss of membrane permeability.

This study unveils early molecular events triggered by and concomitant with the ablation of *Ranbp2* in cone photoreceptors, including changes in *Mmp11*, *Crx* and *CoREST* expressions at P7 and of MMP11 activity at P13, followed by the activation of caspase 7 and a compensatory burst in the expression of the MMP11 substrate, *Cola6α3*, at P20, when the degeneration of cones is well underway. These events are accompanied by the up-regulation of cone survival genes, such as genetic variants of *miR-124a* and its downstream target transcript, *Lhx2*, at P13, when the down-regulation of cone-specific genes, such as M- and S-opsins, becomes significant. *miR-124* promotes the translational suppression of *Lhx2*, a process which has been linked to the suppression of apoptosis during cone photoreceptor differentiation and aberrant sprouting of hippocampal neurons [73]. In contrast to these observations, up-regulation of *miR-124a* does not prevent the demise of mature cone photoreceptors and occurs concomitantly with a rise of *Lhx2* levels. Further, changes in *CoREST* levels, a target of *miR-124* [79], preceded those of all *miR-*124 variants. These observations suggest that the *miR-124* variants may act on other substrates, such as *Foxa2* [90], and that may modulate the survival of cones (or rods) independently of Lhx2 and CoREST suppression [79]. Finally, even though cones account for only ∼3% of all photoreceptor types of the mouse retina [59], the return of cone-derived caspase 7 and MMP11 activities and rod-derived caspase 3 activity to control levels after cones have degenerated strongly support these events are triggered solely by the Ranbp2-dependent dysfunction of cones.

The early and selective activation of MMP11 followed by the development of prominent interstitial spaces between photoreceptor cell bodies, swelling of the lower fibers of cones, and the loss of membrane permeability of rods supports that MMP11 plays an important role in the development of autonomous and non-autonomous photoreceptor degeneration. These roles may include autocrine, paracrine and even intracrine actions, whereby intracrine and paracrine functions contribute to intracellular erosion of cones and non-autonomous death of rods, respectively. In comparison to other MMP proenzymes, MMP11 shows important functional differences. While most MMPs are activated extracellularly, MMP11 is secreted in the active form [91]. This exposes intracellular substrates to active MMP11, a potential intracrine signal, and its extracellular proteolytic functions may stimulate autocrine and/or paracrine signaling. Regardless of its molecular mechanisms of action(s), increased levels of MMP11 expression is reported to modulate cell survival [92]–[96], to act as a negative prognostic of cancer patient survival [97]–[99], and to promote oncogenic homing, tumorigenesis and metastasis [92], [96], cardinal manifestations triggered also by haploinsufficiency and hypomorphism of *Ranbp2* [48].

MMP11 up-regulation has been reported to promote the suppression of apoptotic and necrotic cell death, rather than stimulation of cell proliferation [95], [96]. These cellular manifestations appear at odds with the physiological phenotypes of our study. It is possible that the distinct pathological outcomes produced by the induction of MMP11 upon loss of Ranbp2 reflect intrinsically distinct tissue/cell-type-dependent signaling cues produced by MMP11 substrates. Hence, etiological distinct disorders, such as cancer and neurodegeneration, may share pathomechanisms with distinct clinical outcomes. The identification of pathophysiological substrates of MMP11 will be critical to uncover the scope of its pathobiological roles and aid toward the design of tissue-selective MMP11 inhibitors. It will be interesting to explore whether paracrine factors and players within the network uncovered by the IPA presented in this work, such as Wnt2b and BDNF, their receptors or nuclear transcriptional factors, are substrates of MMP11 or exert regulatory effects on MMP11 expression or activity. Although a specific pharmacological inhibitor of MMP11 is not available [100], [101], the genetic interactions between *Mmp11* and *Ranbp2* may be revealed by assessing the effect of genetic ablation of *Mmp11* on the pathophysiological manifestations observed in *HRGP-cre:Ranbp2^−/−^* (e.g. loss of membrane permeability of rods and rod apoptosis) and to define a potential role for MMP11 in neuroprotection against autonomous and non-autonomous cell death mechanisms affecting cone or rod photoreceptor survival.

Another critical outcome of this work is the finding of non-autonomous apoptotic death of rod (Nr2E3^+^) photoreceptors upon cone dysfunction and death. TUNEL^+^ cones were never identified at any age, while dying rods comprised a mixed population of TUNEL^+^, EthD-III^+^ or both. Thus, rods undergo a programmed cell death that is triggered by the demise of cones. Important, rod cell death ceases at P27, when cone degeneration is complete based on the lack of apoptotic bodies, the absence of Parp1^+^ and caspase3/7^+^ cells, and the preservation of the ONL and rod photoreceptor function at P27 and later ages.

Our results also indicate the presence of atypical mechanisms of cell death. While classical cell death features were identified during cone or rod degeneration, the following observations do not match previously recognized canonical paradigms. First, caspase 7 activation, typically observed in apoptosis, was found in dying cones lacking cleaved Parp1, a substrate of caspase 7. Instead, cone death was characterized by the rampant disintegration of critical subcellular structures, such as outer segments and mitochondria, swelling of lower fibers and cone pedicles, features that are consistent with necrosis. However, EthD-III^+^ cones were not identified as would be expected from the typical loss of membrane permeability caused by necrosis. Second, although rod photoreceptor death is consistent with apoptosis as supported by the presence of caspase 3^+^, Parp1^+^ and TUNEL^+^-cell bodies, classic necrotic features were also present, including a loss of plasma membrane permeability (EthD-III^+^ cell bodies) and limited formation of electron lucent areas in the matrix of mitochondria at synaptic spherules. These observations indicate that activation of caspase 3 and caspase 7 does not determine the mode of rod or cone cell death, respectively. This conclusion is supported by comparable rates of rod death in *rd1* mice on a wild-type or *caspase3^−/−^* background [43]. Third, we did not observe a release of cytochrome C and AIF to the cytoplasm, as typified in apoptosis [66] and observed in other photoreceptor degeneration models [35], [45], an induction of RIP1, RIP3, or caspase 8 activation as observed in necroptosis [61], [69] and lipidation of microtubule-associated protein 1 light chain 3 (LC3/Atg8) that is typical of autophagy [70], [71]. Altogether these data support the existence of complex, shared, unique and thus atypical cell death mechanisms between rod and cone photoreceptors. These manifestations are likely determined by the cell-type dependent pleiotropic molecular and metabolic activities of Ranbp2 [47]–[54]. Emerging mouse models of *Ranbp2* harboring losses in selective domains and functional activities of Ranbp2 will aid in parsing the contribution of such activities to cellular functions and intrinsic or extrinsic cell death modalities unique or shared by several diseases.

Finally, our data show that the non-autonomous death of rods upon loss of Ranbp2 in cones is not promoted by the absence of cones or loss of rod-cone cell contacts. Instead, a likely mechanism is the release of a diffusible factor by cone photoreceptors upon their dysfunction that is deleterious to rod photoreceptors. MMP11 activation is an excellent candidate to play a critical role in such paracrine and death signaling. Our data predict that the topographic density of cone and rod photoreceptors in the retina will play a determinant role in the level of rod photoreceptor degeneration. This issue is of crucial significance to human retinal dystrophies, because the unique and heterogeneous topographic distributions of photoreceptor types across the human retina often mirror retinal pathologies with regional tissue and clinical hallmarks, such as RP, cone-rod dystrophies and age-related macular degeneration (AMD). It is likely that loss of biological activities regulated by RANBP2 will exert prominent pathological outcomes in regions of the retina, such as the macula, where the topographic density of cone and rod photoreceptors is similar.

## Materials and Methods

### Animals

HRGP-Cre mice (kindly provided by Yun-Zheng Le, University of Oklahoma Health Sciences Center) [56], [57] were crossed to *Ranbp2^+/flox^* mice (kindly provided by Jan M. van Deursen, Mayo Clinic College of Medicine) [48] to produce *HRGP-cre:Ranbp2^+/flox^*. *HRGP-cre:Ranbp2^+/flox^* were then crossed to *Ranbp2^+/flox^* or *Ranbp2^Gt(pGT0pfs)630Wcs/+^* [47] to generate *HRGP-cre:Ranbp2^+/−^* (+/−) and *HRGP-cre:Ranbp2^−/−^* (−/−). Mice were in the following mixed genetic background: 129 SvJ,C57BL/6J,FVB/N,129olaHsd. Mice were screened also for *rd1* and *rd8* alleles. Mice were raised in a pathogen-free transgenic barrier facility at <70 lux and given *ad libitum* access to water and chow diet 5LJ5 (Purina, Saint Louis, MO). Animal protocols were approved by the Institutional Animal Care and Use Committees at Duke University and Cleveland Clinic, and all procedures adhered to the ARVO guidelines for the Use of Animals in Vision Research.

### Antibodies

Primary antibodies used this study were: mouse anti-Cre (Covance, Princeton, NJ), rabbit anti-Cre (Novagen, Gibbstown, NJ), rabbit anti-Arr4 (Millipore/Pel-Freez Biologicals , Billerica, MA), rabbit anti-L/M opsin (#Ab 21069) [55], rabbit monoclonal anti-cleaved caspase3 Asp175 (Cell Signaling, Danvers, MA), rabbit polyclonal anti-cleaved-caspase 7 Asp353 (Cell Signaling), rabbit anti-cleaved caspase 8 Asp391 (Cell Signaling), rabbit anti-cleaved caspase 9 Asp 330 (Cell Signaling), rabbit anti-cleaved Parp1 Asp214 (Cell Signaling), mouse anti-full-length Parp1 (BD Bioscience, San Jose, CA), mouse anti-LC3 B (Cell Signaling, Danvers, MA), mouse anti-cytochrome C (BD Bioscience, San Jose, CA), mouse anti-mitochondrial heat shock protein 70 (Affinity Bioreagent, Golden, CO), rabbit-cytosolic heat shock protein 70 (Assay Design, Farmingdale, NY), rabbit anti-GFAP (DAKO, Carpinteria, CA), mouse anti-glutamine synthase (Sigma Aldrich, Saint Louis, MO), mouse anti-RIP1 (BD Bioscience, San Jose, CA), rabbit anti-RIP3 (Sigma Aldrich, Saint Louis), rabbit anti-GAPDH and goat anti-AIF1 (Santa Cruz Biotechnology, Santa Cruz, CA), rabbit anti-Apaf-1 (LSBio, Seattle, WA), mouse anti PSD-95 (Affinity BioReagent), mouse anti-MMP11(Thermo Scientific, Waltham, MA), rabbit anti-S-opsin (Millipore/Pel-Freez Biologicals), rabbit anti-Nr2E3 (Proteintech, Hayward, CA) and Peanut Agglutinin TRTIC conjugate (Sigma-Aldrich). Alexa-conjugated secondary antibodies (408, 488, 568 and Cy5) were from Invitrogen (Carlsbad, CA).

### RT-PCR and qRT-PCR

For total RNA and pre-miRNA isolation, retinas were homogenized with the TRIZOL Regent (Invitrogen) using Bullet Blender BBX24 (Next Advance Inc., Averill Park, NY) in the presence of 0.5 mm zirconium oxide beads (Next Advance Inc., Averill Park, NY) for 3 min at 8,000 rpm. RNA was reverse transcribed into cDNA using SuperScript II reverse transcriptase (Invitrogen). For RT-PCR, the ∼380 bp amplicon encompassing fused exons 1 and 3 of recombinant *Ranbp2* mRNA was amplified from 250 ng of retinal cDNA. PCR was performed using primer 1 (Pr1:CGCCCCGAGAGTACATTTCTA) and primer 2 (Pr2:AAGTTTATTCCATCCATCTTCA) with GoTaq Green Master Mix (Promega, Madison, WI) under the following cycling conditions: 5 min/95°C of initial denaturation, 35 cycles/94°C (30 s), 55°C(30 s) and 72°C(30 s) with final elongation step at 72°C for 3 mins. Same cycling conditions were applied for *Cre* (CTAATCGCCATCTTCCAGCAGG, AGGTGTAGAGAAGGCACTTAGC) and *Gapdh* (GCAGTGGCAAAGTGGAGATT, GAATTTGCCGTGAGTGGAGT). qRT-PCR reactions were carried out with 8 ng of cDNA, 800 nM forward and reverse primers, 10 µl of 2×SYBR® Green PCR Master Mix (Applied Bioscience, Warrington, MA) in a 20 µl final volume in 48-well plates using the ECO™ Real-Time PCR system (Illumina, San Diego, CA). The relative amount of transcripts was calculated by the ΔΔ C_T_ method using *Gapdh* as reference (*n = 3–4*). Primer sequences and designations are provided in Table S1.

### Immunohistochemistry

The superior region of mouse cornea was burned using Low Temperature Cautery (Bovie Medical Corporation, St. Peterburg, FL) immediately after mice were killed. For immunohistochemistry, eyeballs were removed and fixed with 2% paraformaldehyde/phosphate-buffer saline (PBS), pH 7.4 for 4 hr after small incisions were made in the anterior portion. Upon removal of the lens, eyecups were infiltrated with 5% sucrose/100 mM PBS, pH 7.4, for 5 hr followed by 30% sucrose/100 mM PBS for 12 hours, embedded in Tissue-Tek O.C.T. compound (Sakura, Torrance) and stored at −80°C. 12 µm thick retinal cryosections along the vertical meridian of the eyecup were mounted on glass slides. For flat mounts, retinas were removed from fixed eyeballs, cut in a four-quadrant cloverleaf pattern using the caruncle as an orientation landmark and fixed for an additional 15 min in a 24-well plate. Specimens were incubated in blocking buffer (PBS, pH 7.4, containing 0.1% Triton X-100, 10% normal goat serum) for 1 hr at RT (for sections) or 12 hr at 4°C (flat mounts), followed by incubation with primary antibodies in incubation buffer (PBS, pH 7.4, containing 0.1% Triton X-100, 5% normal goat serum) overnight at RT (for sections) or for 3 days at 4°C (flat mounts). Specimens were washed thrice with washing buffer (PBS/0.1% Triton X-100) for 10 min, and incubated in incubation buffer for 2 hr with Alexa-conjugated secondary antibodies (1∶1,000; Invitrogen). For Nr2E3 immunostaining, eyeballs were fixed instead with 1% paraformaldehyde for 1 hr at room temperature. 6 µm-thick retinal cryosections were incubated in blocking buffer for 1 hr at room temperature followed by treatment with proteinase K (20 µg/ml, Promega, Madison, WI) for 9 min and standard immunostaining protocols as described earlier. Specimens were washed again thrice and mounted on glass slides for visualization and image acquisition. Images were acquired with a Nikon C1^+^-laser scanning confocal microscope coupled with a LU4A4 launching base of 4-solid state diode lasers (407 nm/100 mW, 488 nm/50 mW, 561 nm/50 mW, 640 nm/40 mW) and controlled by the Nikon EZC1.3.10 software (v6.4).

### TUNEL assay

TUNEL assays were performed with the DeadEnd Fluorometric TUNEL System (Promega, Medison) with the following modifications from the manufacturer's instructions. Briefly, specimens were incubated with 20 µg/ml proteinase K for 15 min at RT followed by fixation with 4% paraformaldehyde for 15 min, and incubations with primary and secondary antibodies along with DAPI (Invitrogen, Carlsbad, CA). Specimens were equilibrated for 5 min in manufacturer's buffer before undergoing TdT reactions for 60 min at 37°C. Reactions were stopped with 2×SSC, washed three times with PBS and mounted.

### EthD-III staining

Fresh P20 retinal explants were incubated with 5 µM EthD-III (Biotium, Hayward, CA) in the Neurobasal^−^A Medium/B-27 Supplement (Invitrogen) for 4 hr at 37°C in a humidified 5% CO_2_ atmosphere, washed vigorously 3 times with 100 mM PBS for 10 min and fixed with 2% paraformaldehyde/100 mM PBS for 20 min at room temperature. Specimens were then processed as described for immunostaining and TUNEL procedures.

### Morphometric analyses

Morphometric analyses of M-cone photoreceptors were performed from 127×127 µm image fields captured with a Nikon C1^+^-laser scanning confocal microscope. Optical slices were 3D-reconstructed for the whole length of outer segments (∼25 µm, step size of 0.5 µm) from retinal flat mounts immunostained with an L/M-opsin antibody [55]. M-cone photoreceptors and the length of their outer segments were then tallied and measured from three image fields for each retina with the post-acquisition Nikon Elements AR (ver3.2) software. Cre^+^, c-casp3^+^ and TUNEL^+^-cells bodies were imaged from flat mounts and three image fields of 127×127 µm and collapsed for the whole depth of ONL (∼60 µm, step size of 3 µm) from the central or peripheral retinal regions. For TUNEL^+^, EthD-III^+^ and Arr4^+^-cells bodies, three image fields of 127×127×5 µm from retinal flat mounts were randomly selected and quantitatively analyzed. For tallying of DAPI^+^, TUNEL^+^,^−^Nr2E3^+^or Cre^+^-cell bodies, whole vertical meridian sections were counted and averaged to generate pie graphs (Excel, Microsoft, Seattle, WA). The distal region of the ONL was arbitrarily defined as the 15 µm distal segment of the ONL up to the external limiting membrane and the remaining segment was defined as proximal. 3D-reconstruction of collapsed images and morphometric analysis was performed with Nikon Elements AR software (ver. 3.2). Two-tailed equal or unequal variance t-test statistical analysis was performed. p≤0.05 was defined as significant.

### SDS-PAGE and immunoblots

Retinas were homogenized at 4°C with Bullet Blender BBX24 (Next Advance Inc., Averill Park, NY) in the presence of 0.5 mm zirconium oxide beads (Next Advance Inc.) and RIPA buffer containing complete protease inhibitors (Roche Applied Bioscience, Penzberg, Germany) and 10 mM iodoacetamide (Sigma Aldrich). Protein concentration was measured by the BCA method using BSA as a standard. Samples (55 µg) were resolved on 11% SDS-polyacrylamide gel electrophoresis (SDS-PAGE), immunoblotted and developed using the SuperSignal Pico West (Thermo Scientific) as described previously [50]. Blots were probed with mouse anti-MMP-11 antibody (Thermo Scientific). Densitometry analysis of immunoblots was performed with Metamorph v7.0 (Molecular Devices, Sunnyvale, CA). Two-tail *t*-test statistical analysis was performed for validation of significant change (p<0.05).

### Caspases' assays

Retinas were homogenized using BulletBlender BBX24 (Next Advance Inc.) with 0.5 mm zirconium oxide beads (Next Advance Inc.) and RIPA buffer. Retinal homogenates were centrifuged at 10,000 g for 15 min at 4°C. Supernatants were collected and protein concentrations determined by the BCA method using BSA as standard. Retinal homogenates were diluted in caspase assay buffer and caspase profiling assays were performed with the Sensolyte AFC Caspase sampler kit according to company's protocol (AnaSpec, Fremont, CA). The following caspase substrates were used for screening: Ac-YVAD-AFC (SB1) and Ac-WEHD-FAC (SB2) for caspase 1, Ac-VDVAD-FAC (SB3) for caspase 2, Ac-IETD-FAC, (SB4) for caspase 8, Ac-DEVD-FAC (SB5) and Z-DEVD-AFC (SB6) for caspases 3/7, Ac-LEHD-FAC (SB7) for caspase 9 and Ac-VEID-AFC (SB8) for caspase 6. Assays were first optimized for substrate dilution, extract concentration and reaction time. Substrate screenings were carried with three different protein concentrations. Analytical assays of caspase 3/7 (SB5) were performed with diluted 50 µl of retinal homogenate (280 ng) and 50 µl of substrate (1∶200 dilution in caspase assay buffer), mixed in the 96 well-plate, incubated with shaking for 1 hr under dark at room temperature. Measurements of fluorescence were performed at excitation/emission/cutoff = 380/500/495 nm with SpectraMax M5 (Molecular Devices, Sunnyvale, CA). Control reactions without extracts were subtracted from the samples' readings.

### Matrix metalloproteinases' assays

MMPs' screening assays were performed with Sensolyte 520 generic MMP assay kit per company's protocol (AnaSpec, Fremont, CA). Retinas extracts were prepared as described previously with the exception that RIPA buffer was replaced with MMP assay buffer (AnaSpec). Briefly, MMP assays are based on the dequenching of fluorescence intensity of 5-FAM upon proteolytic cleavage of 5-FRAM/QXL520 peptide substrate by MMPs. MMPs screenings, except MMP11, were performed upon activation with 1 mM 4-aminophenylmercuric acetate (APMA) at different time intervals (MMP2, 7, 8 and 13: 1 hr activation; MMP9, 12 and 14: 2 hr activation; MMP1: 3 hr activation; MMP3 and 10: 24 hr activation) at 37°C followed by 1 hr incubation with 5-FRAM/QXL520 peptide substrate. For MMP11, retinal extract was directly mixed with substrate and incubated for 1 hr before fluorescence reading. 50 µl of activated/non-activated protein extract at 56, 112 or 224 ng and 50 µl of substrate (final substrate dilution 1∶200) were mixed in a 96 well-plate, incubated with shaking for 1 h at room temperature at dark. Measurements of fluorescence were performed at excitation/emission/cutoff = 490/520/495 nm with SpectraMax M5 (Molecular Devices, Sunnyvale, CA). Control measurements without retinal extracts under the same conditions were subtracted from the sample readings. Analytical assays with MMP11 were performed with 56 ng of retinal extract.

### Subcellular fractionation

Mitochondrial and cytosolic fractions of the retina were isolated with the Mitochondria Isolation Kit for Tissue (Abcam, Cambridge, MA) per manufacturer's instruction. Briefly, retinas were washed with washing buffer, homogenized with a Kontes Microtube Pellet Pestle Rod with motor in isolation buffer, centrifuged at 1,000× g for 10 mins, the pellet was saved (nuclear-enriched fraction) and supernatant was centrifuged again at 12,000× g for 15 min. The supernatants (cytosolic fraction) were saved, the pellets (mitochondrial fraction) washed with Isolation buffer twice and re-suspended with isolation buffer with complete protein inhibitor cocktail (Roche Applied Bioscience, Penzberg, Germany).

### Semi-thin sections and transmission electron microscopy

Eyeballs were removed and fixed with 2% glutaraldehyde:paraformaldehyde/0.1% cacodylate buffer, pH 7.2, overnight at 4°C. For semi-thin histological sections, 0.5 µm sections along the vertical meridian were mounted on glass slides and stained with 1% methylene blue. Light images of the retina sections were acquired with a Axiopan-2 light microscope controlled by Axovision Rel 4.6 and coupled to a AxioCam HRc digital camera (Carl Zeiss, Germany). For electron microscopy, specimens were post-fixed in 2% osmium tetraoxide in 0.1% cacodylate buffer and embedded in Spurr resin. 60 nm-thick sections were cut with Leica Ultracut S (Leica Microsystems, Waltzer, Germany), stained with 2% uranyl acetate/4% lead citrate and imaged with JEM-1400 transmission electron microscope (JEOL, Tokyo, Japan) coupled with an ORIUS 1000CCD camera.

### Ingenuity pathway analysis

The Ingenuity pathway analysis (IPA, Ingenuity Systems, Redwood City, CA) was used to examine the gene dataset modulated by loss of Ranbp2 and to define connectivity maps and networks. Network of genes are algorithmically produced based on their connectivity. Significant network scores reflect the negative logarithm of a *P* value associated with the likelihood of connectivity of a set of genes in a network. The network with highest score (score of 22) was chosen for further analysis.

### Electroretinography

After overnight dark adaptation, mice were anesthetized (ketamine: 80 mg/kg; xylazine: 16 mg/kg) and eyedrops were used for pupil dilation (1% tropicamide; 2.5% phenylephrine HCl; 1% cyclopentolate HCl) and corneal anesthesia (1% proparacaine HCl). The active electrode was a stainless-steel wire active electrode that contacted the corneal surface through 1% methylcellulose; needle electrodes placed in the cheek and tail served as reference and ground leads, respectively. Responses were differentially amplified (0.3–1,500 Hz), averaged, and stored using a UTAS E-3000 signal averaging system (LKC Technologies, Gaithersburg, MD). Strobe flash stimuli were initially presented in darkness within a ganzfeld bowl. Flash luminance ranged from −3.6 to 2.1 log cd s/m^2^ and stimuli were presented in order of increasing luminance. A steady adapting field (20 cd/m^2^) was then presented in the ganzfeld. After a 7 min light adaptation period cone ERGs were evoked by strobe flash stimuli superimposed upon the adapting field. Flash luminance ranged from −0.8 to 1.9 log cd s/m^2^ and stimuli were presented at 2 Hz in order of increasing luminance. The amplitude of the *a*-wave was measured 8 ms after flash onset from the prestimulus baseline. The amplitude of the *b*-wave was measured from the *a*-wave trough to the peak of the *b*-wave or, if no *a*-wave was present, from the pre-stimulus baseline.

## Supporting Information

Figure S1 Co-expression of S-opsin (a′), M-opsin (b′), Cre (a″, b″) or PNA (a′″, b′″) in the superior (a–a″″) and inferior regions (b–b″″) of the retina of *HRGP-cre:Ranbp2^+/−^* at P20. a″″ and b″″ are overlay images. Note the lack of M-and S-opsin expression in the superior (dorsal) and inferior (ventral) regions of the retina, respectively. Legends: PNA, peanut agglutinin; Cre, cre recombinase. Scale bars = 20 µm.
(PDF)

Figure S2 Isolated Arr4^+^-cone photoreceptor neuron without its outer and inner segment compartments in a 6-week old *HRGP-cre:Ranbp2^−/−^* (−/−) mouse. Scale bars = 25 µm.
(PDF)

Figure S3 Degeneration of S-cone photoreceptors and their outer segments in *HRGP-cre:Ranbp2^−/−^* at P20. (A) Retinal flat mount images of *HRGP-cre:Ranbp2^+/−^* (+/−; a, a′) and *HRGP-cre:Ranbp2^−/−^* (−/−; b, b′) immunostained with an antibody against S-opsin showing severe S-cone cell loss in the inferior (ventral)/central region of the retina of −/− mice. 3D-reconstruction images of retinal flat mount images (a, b) and magnifications of ROI (white box) in “a” and “b” (a′, b′). Note the prominent formation of S-opsin aggregates in the outer segments of S-cone photoreceptors upon their degeneration. (B–C) Quantitative analyses of the number (B) and length of outer segments (C) of S-cone photoreceptors in the inferior/central regions of retina of −/− and +/− mice. Legend: Data shown represent the mean ± SD, *n* = 3; *, *p*<0.01; o, *p*<0.0001. Scale bars = 25 µm (a, b), 5 µm (a′, b′).
(PDF)

Figure S4 Identification of TUNEL^+^ , EthD-III^+^ and TUNEL^+^ EthD-III^+^ in Nr2E3^−^ cell bodies of photoreceptors between *HRGP-cre:Ranbp2^+/−^* (+/−) and *HRGP-cre:Ranbp2^−/−^* mice (−/−) at P20. Representative images of EthD-III/TUNEL/Nr2E3 staining of photoreceptors of +/− (a–a″″) and −/− (b–b″″) showing subpopulations of Nr2E3^−^ -photoreceptor cell bodies in −/− that are Nr2E3^−^ TUNEL^+^ (green arrow), Nr2E3^−^ TUNEL^+^ EthD-III^+^ (yellow arrow) and Nr2E3^−^ EthD-III^+^ (red arrow). Scale bars = 25 µm.
(PDF)

Figure S5 Screening of caspase activities with caspase-selective substrates in retinal extracts of P20 mice. Retinal extracts have strong and increased activities of caspases 3 and 7. Retinal extracts exhibit also mild increases of caspases 8 and 9 activities. Proteolytic activities reflect the activities of *HRGP-cre:Ranbp2^−/−^* relative to *HRGP-cre: Ranbp2* ^+/−^ mice. Legend: RFU, relative fluorescent units; casp, caspase.
(PDF)

Figure S6 qRT-PCR of transcriptional regulation of *caspase 3* (*casp3*) and *caspase 7* (*casp7*) in retinas of *HRGP-cre:Ranbp2^−/−^* compared to *HRGP-cre:Ranbp2* ^+/−^ mice. There is a sequential and transient up-regulation of *caspase 3* and *caspase 7* at P13 and P20, respectively, in *HRGP-cre:Ranbp2^−/−^*. Legend: Data shown represent the mean ± SD, *n* = 3; *, *p*<0.05; **, *p*<0.01.
(PDF)

Figure S7 (A) Immunohistochemistry of retinal sections showing the localizations of cleaved Parp1 (c-Parp1) (a′–b′), TUNEL^+^ (a″, b″) and Cre^+^ (a′″, b′″), and cleaved caspase 9 (c-casp9) (c′, d′) , TUNEL^+^ (c″, d″) and Cre^+^ (c′″, d′″), in photoreceptor cell bodies of *HRGP-cre:Ranbp2* ^+/−^ (+/−; b–b″″, d–d″″) and *HRGP-cre:Ranbp2^−/−^* (−/−; a–a″″, c–c″″) at P9 of age. a″″–d″″ are overlay images. Note that there were no c-Casp9^+^-photoreceptor cell bodies in −/− at P13 and P20 (data not shown). White arrow head indicates c-Casp9^+^ or c-Parp1^+^ cell. Sections were counterstained with DAPI (a–d). Scale bars = 25 µm. (B) qRT-PCR of transcriptional regulation of *caspase 9*, *Parp1* and *caspase 8* in retinas of *HRGP-cre:Ranbp2^−/−^* compared to *HRGP-cre:Ranbp2* ^+/−^ mice. There was selective up-regulation of *caspase 9* and *Parp1*, but not of *caspase 8*, in *HRGP-cre:Ranbp2^−/−^* mice. Legend: Data shown represent the mean ± SD, *n* = 3–4; *, *p*<0.05; **, *p*<0.01.
(PDF)

Figure S8 Lack of activation of canonical apoptotic, necroptotic and macroautophagic death upon ablation of *Ranbp2* in cones at P13. (A) Subcellular fractionations of mitochondria (M) and cytosol (C) of retinal homogenates shows that there are no traces of AIF and cytochrome C (cyt C) release from the M to C fraction in either *HRGP-cre:Ranbp2* ^+/−^ (+/**−**) or *HRGP-cre:Ranbp2^−/−^* (−/−) mice . GAPDH is a loading control. mHsp70 and GADPH are mitochondrial and cytosolic markers, respectively. (B) The levels of the apoptosis-inducing factor (AIF) are not changed in the nuclear-enriched fraction of either genotype . (C) The levels of receptor interacting proteins 1 and 3 (RIP1 and RIP3) remain unchanged between *HRGP-cre:Ranbp2* ^+/−^ (+/−) and *HRGP-cre:Ranbp2^−/−^* (−/−) mice. Graph on the left is a quantification of immunoblot on the right. Hsc70 is a loading control. (D) The ablation of *Ranbp2* in cone photoreceptors does not cause the generation of autophagosomal LC3B II, whereas ectopic expression of Ranbp2 in COS7 cells produces LC3B II (control). GAPDH is a loading control. Legend: mHsp70, mitochondrial heat shock protein 70; Hsc 70, cytosolic heat shock protein 70; GADPH, glyceraldehyde 3-phosphate dehydrogenase, LC3B II, autophagosomal membrane-associated light chain 3 II isoform; LC3B I, cytosolic light chain 3 II isoform; +/−, *HRGP-cre:Ranbp2* ^+/−^, −/−, *HRGP-cre:Ranbp2^−/−^*; Data shown represent the mean ± SD, *n* = 3–5; ns, not significant (p>0.05).
(PDF)

Figure S9 Selective activation and up-regulation of MMP11 upon ablation of *Ranbp2* in cone photoreceptors. (A) Screening for activation of MMPs upon ablation of *Ranbp2* in cones at P20. There is a selective activation of MMP11, but not of any other MMPs tested. In inset graph, the activity of MMP11 was normalized to control reactions without retinal extracts. *HRGP-cre:Ranbp2^−/−^* (−/−) compared to *HRGP-cre:Ranbp2* ^+/−^ (+/−) mice have ∼3-fold increase of MMP11 activity. (B) qRT-PCR shows the transient down-modulation of *Mmp11* at P7 followed by its sustained up-regulation until P20 in *HRGP-cre:Ranbp2^−/−^*. By contrast, the levels of *Timp3* remain largely unchanged, except at P9, when there is a rise of its levels. Legends: *Timp3, tissue inhibitor metalloproteinase 3*; RFU, relative fluorescence units. Data shown represent the mean ± SD, *n* = 3–4.
(PDF)

Figure S10 Localization of postsynaptic density 95 (PSD95) in *HRGP-cre:Ranbp2^−/−^* (−/−) compared to *HRGP-cre:Ranbp2^+/−^* (+/−). Immunolocalization of postsynaptic density marker, PSD-95, in retinas of +/− (a–a″″) and −/− mice (b–b″″) at P20. No changes of PSD95 were discerned between −/− and +/−. Legends: PSD-95, postsynaptic density protein 95, Arre4, cone arrestin 4, PNA, Peanut Agglutinin. Scale bars = 25 µm.
(PDF)

Figure S11 Absence of extended rod photoreceptor degeneration in *HRGP-cre:Ranbp2^−/−^* (−/−). (A) The quantification of the degeneration of rod photoreceptors by measuring the outer nuclear layer (ONL) thickness along the vertical meridian of the eye. There were no significant changes in ONL thickness between −/− and +/− from P9 to 3-month old mice. Plots demonstrate ONL thickness of at P9, P13, P20, P27 and 3-month of age. (B) Histograms of the average ratio of the ONL thickness between −/− and +/− from P9 to 3-month of age (p>0.05). (C) Comparison of photoreceptor cell body density in the ONL between −/− and +/− at P20. There were no significant differences. Nuclei in the ONL were counted and normalized per area from light microscopy images of methylene blue stained and semi-thin retinal sections. Legend: ONH, Optic nerve head, ONL, Outer nuclear layer. Data shown represent the mean ± SD, *n* = 3; n.s., not significant (p>0.05).
(PDF)

Table S1 Designations and sequences of primers used for gene expression analyses by qRT-PCR. List of primers used for gene expression profiling of *HRGP-cre:Ranbp2^−/−^* and *HRGP-cre:Ranbp2* ^+/−^ mice by qRT-PCR.
(PDF)
