# Supplementary figures and images for: Distinct and Atypical Intrinsic and Extrinsic Cell Death Pathways between Photoreceptor Cell Types upon Specific Ablation of Ranbp2 in Cone Photoreceptors
Source: PLoS Genet. 2013 Jun 20;9(6):e1003555. doi: 10.1371/journal.pgen.1003555 (PMC3688534; doi:10.1371/journal.pgen.1003555)

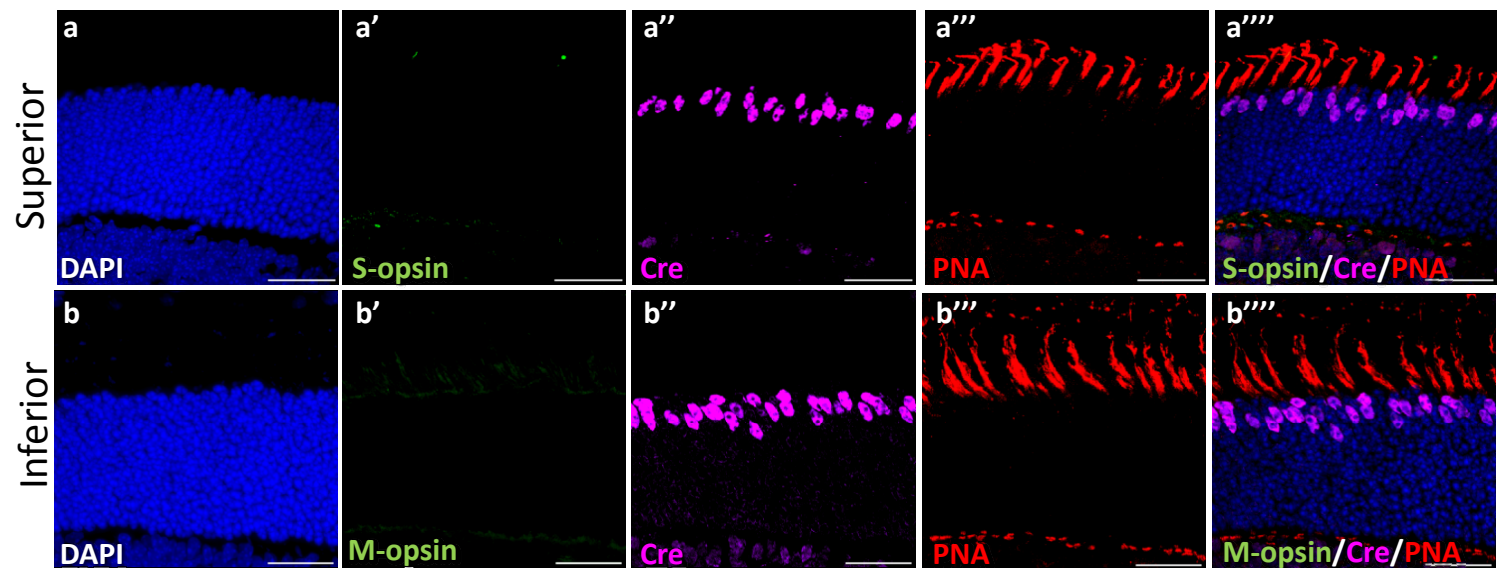

Supplement: Figure S1 — Co-expression of S-opsin (a′), M-opsin (b′), Cre (a″, b″) or PNA (a′″, b′″) in the superior (a–a″″) and inferior regions (b–b″″) of the retina of HRGP-cre:Ranbp2+/− at P20. a″″ and b″″ are overlay images. Note the lack of M-and S-opsin expression in the superior (dorsal) and inferior (ventral) regions of the retina, respectively. Legends: PNA, peanut agglutinin; Cre, cre recombinase. Scale bars = 20 µm. (PDF) [file pgen.1003555.s001.pdf]

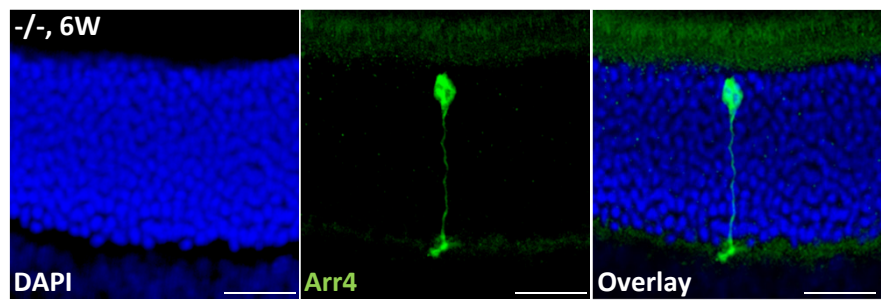

Supplement: Figure S2 — Isolated Arr4+-cone photoreceptor neuron without its outer and inner segment compartments in a 6-week old HRGP-cre:Ranbp2−/− (−/−) mouse. Scale bars = 25 µm. (PDF) [file pgen.1003555.s002.pdf]

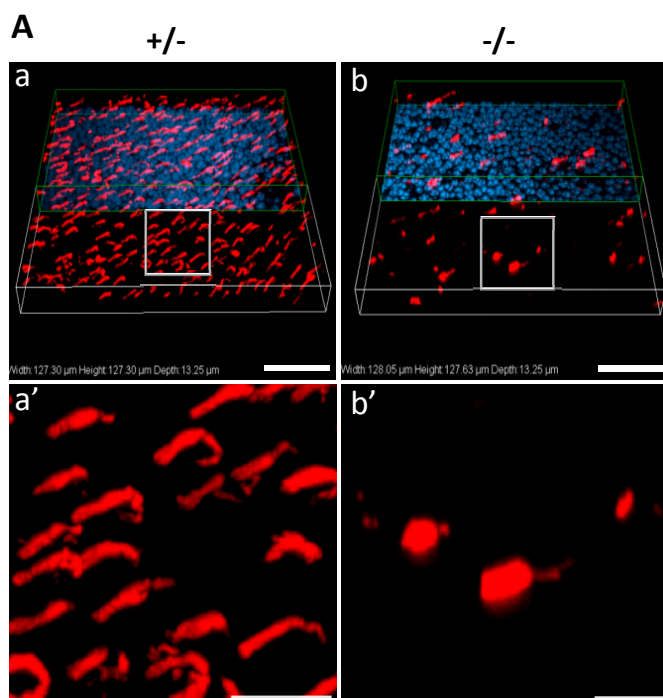

**B**

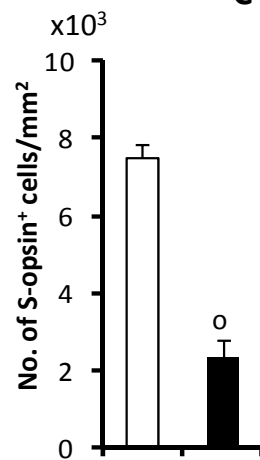

**C**

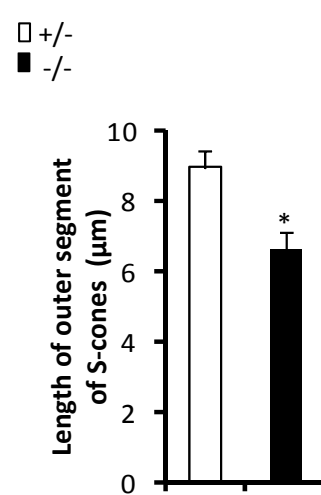

Supplement: Figure S3 — Degeneration of S-cone photoreceptors and their outer segments in HRGP-cre:Ranbp2−/− at P20. (A) Retinal flat mount images of HRGP-cre:Ranbp2+/− (+/−; a, a′) and HRGP-cre:Ranbp2−/− (−/−; b, b′) immunostained with an antibody against S-opsin showing severe S-cone cell loss in the inferior (ventral)/central region of the retina of −/− mice. 3D-reconstruction images of retinal flat mount images (a, b) and magnifications of ROI (white box) in “a” and “b” (a′, b′). Note the prominent formation of S-opsin aggregates in the outer segments of S-cone photoreceptors upon their degeneration. (B–C) Quantitative analyses of the number (B) and length of outer segments (C) of S-cone photoreceptors in the inferior/central regions of retina of −/− and +/− mice. Legend: Data shown represent the mean ± SD, n = 3; *, p<0.01; o, p<0.0001. Scale bars = 25 µm (a, b), 5 µm (a′, b′). (PDF) [file pgen.1003555.s003.pdf]

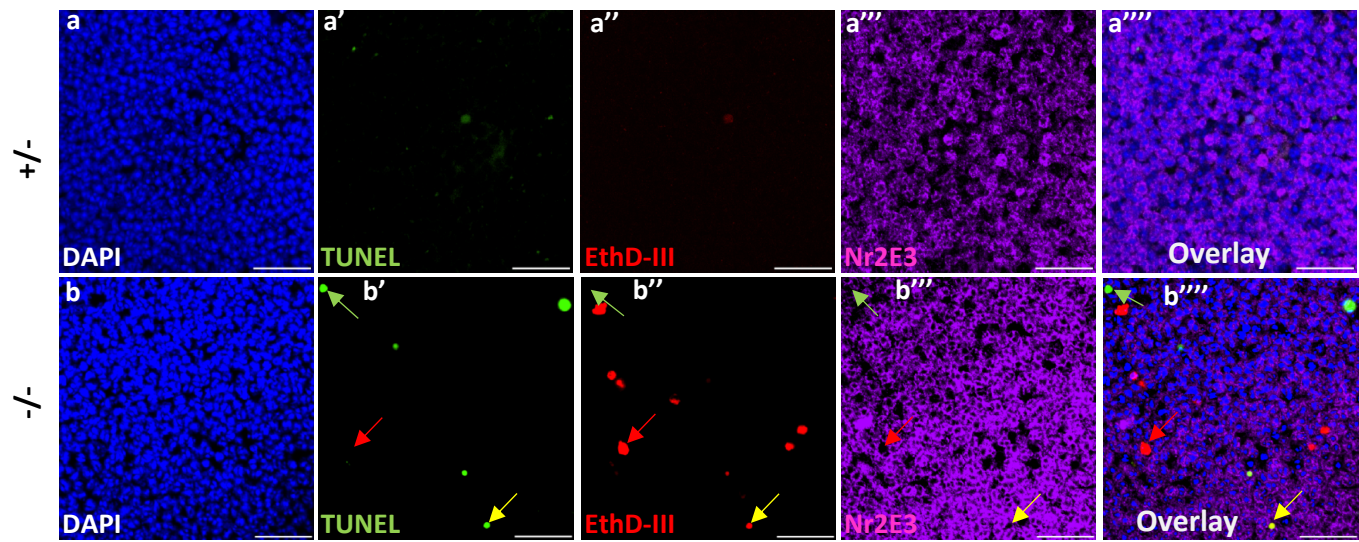

Supplement: Figure S4 — Identification of TUNEL+ , EthD-III+ and TUNEL+ EthD-III+ in Nr2E3− cell bodies of photoreceptors between HRGP-cre:Ranbp2+/− (+/−) and HRGP-cre:Ranbp2−/− mice (−/−) at P20. Representative images of EthD-III/TUNEL/Nr2E3 staining of photoreceptors of +/− (a–a″″) and −/− (b–b″″) showing subpopulations of Nr2E3− -photoreceptor cell bodies in −/− that are Nr2E3− TUNEL+ (green arrow), Nr2E3− TUNEL+ EthD-III+ (yellow arrow) and Nr2E3− EthD-III+ (red arrow). Scale bars = 25 µm. (PDF) [file pgen.1003555.s004.pdf]

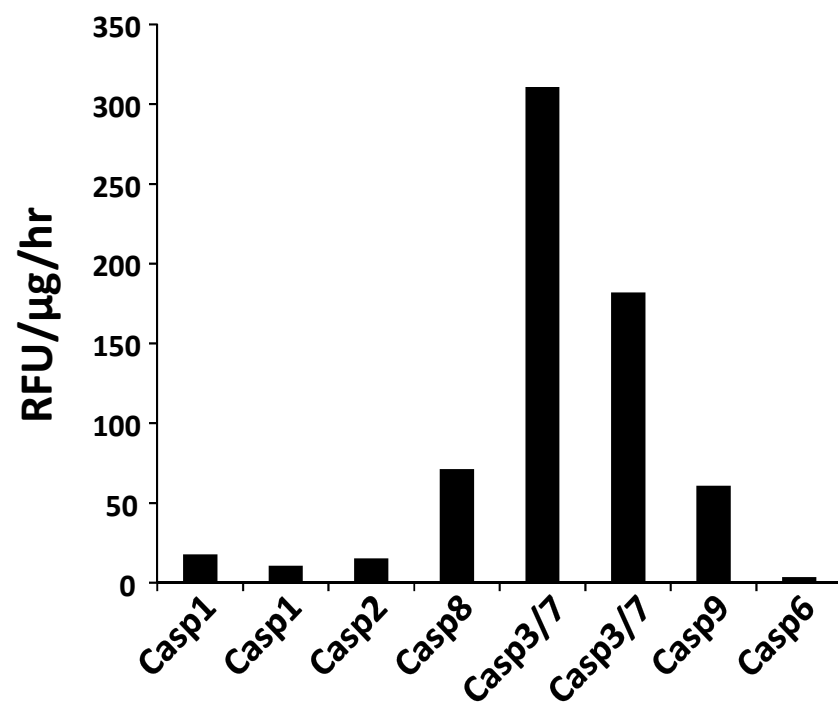

Supplement: Figure S5 — Screening of caspase activities with caspase-selective substrates in retinal extracts of P20 mice. Retinal extracts have strong and increased activities of caspases 3 and 7. Retinal extracts exhibit also mild increases of caspases 8 and 9 activities. Proteolytic activities reflect the activities of HRGP-cre:Ranbp2−/− relative to HRGP-cre: Ranbp2 +/− mice. Legend: RFU, relative fluorescent units; casp, caspase. (PDF) [file pgen.1003555.s005.pdf]

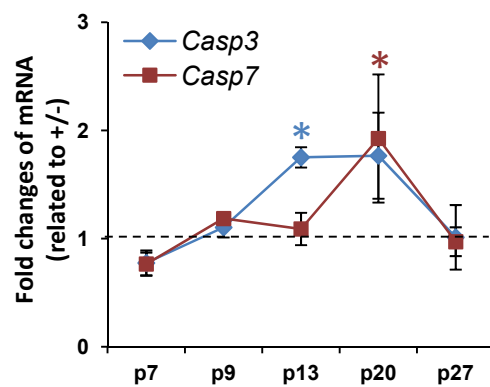

Supplement: Figure S6 — qRT-PCR of transcriptional regulation of caspase 3 (casp3) and caspase 7 (casp7) in retinas of HRGP-cre:Ranbp2−/− compared to HRGP-cre:Ranbp2 +/− mice. There is a sequential and transient up-regulation of caspase 3 and caspase 7 at P13 and P20, respectively, in HRGP-cre:Ranbp2−/−. Legend: Data shown represent the mean ± SD, n = 3; *, p<0.05; **, p<0.01. (PDF) [file pgen.1003555.s006.pdf]

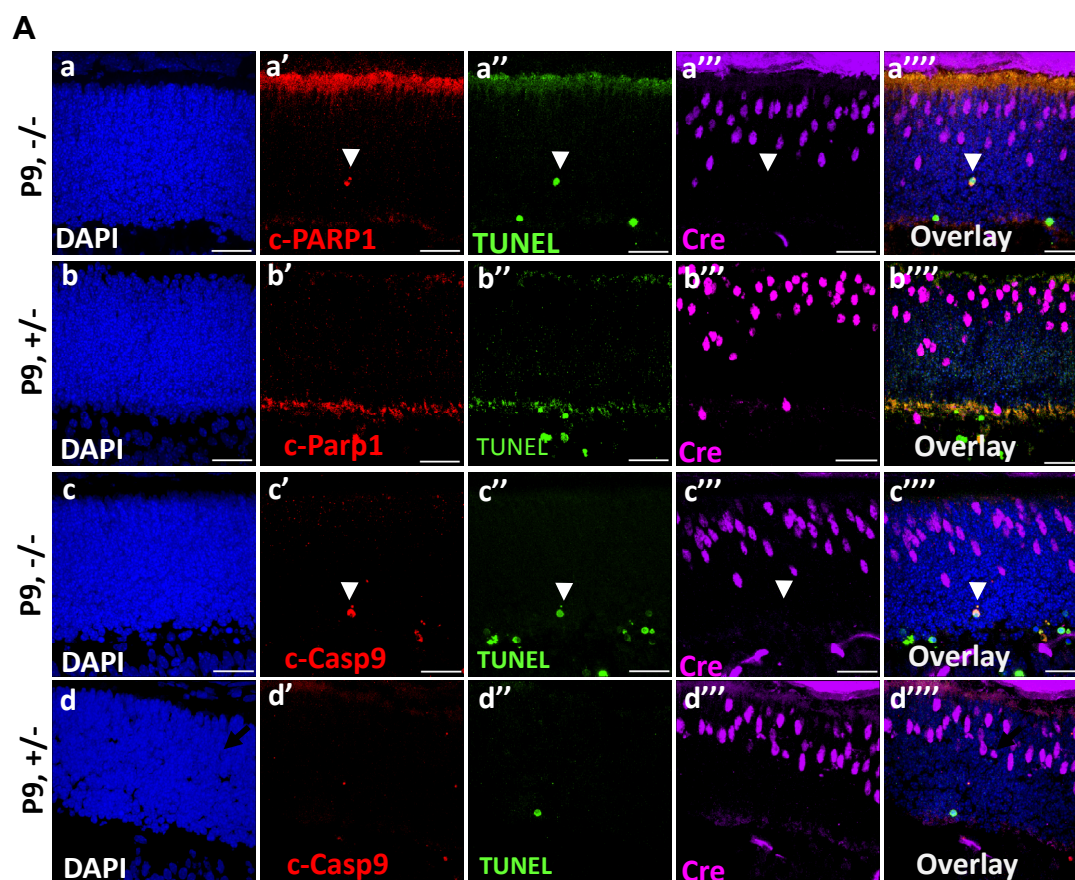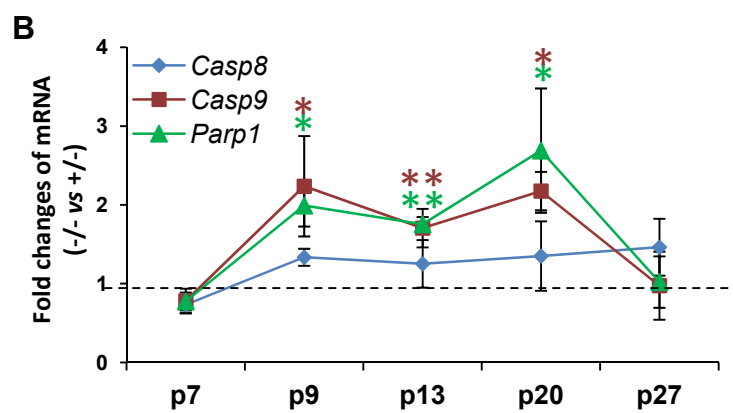

Supplement: Figure S7 — (A) Immunohistochemistry of retinal sections showing the localizations of cleaved Parp1 (c-Parp1) (a′–b′), TUNEL+ (a″, b″) and Cre+ (a′″, b′″), and cleaved caspase 9 (c-casp9) (c′, d′) , TUNEL+ (c″, d″) and Cre+ (c′″, d′″), in photoreceptor cell bodies of HRGP-cre:Ranbp2 +/− (+/−; b–b″″, d–d″″) and HRGP-cre:Ranbp2−/− (−/−; a–a″″, c–c″″) at P9 of age. a″″–d″″ are overlay images. Note that there were no c-Casp9+-photoreceptor cell bodies in −/− at P13 and P20 (data not shown). White arrow head indicates c-Casp9+ or c-Parp1+ cell. Sections were counterstained with DAPI (a–d). Scale bars = 25 µm. (B) qRT-PCR of transcriptional regulation of caspase 9, Parp1 and caspase 8 in retinas of HRGP-cre:Ranbp2−/− compared to HRGP-cre:Ranbp2 +/− mice. There was selective up-regulation of caspase 9 and Parp1, but not of caspase 8, in HRGP-cre:Ranbp2−/− mice. Legend: Data shown represent the mean ± SD, n = 3–4; *, p<0.05; **, p<0.01. (PDF) [file pgen.1003555.s007.pdf]

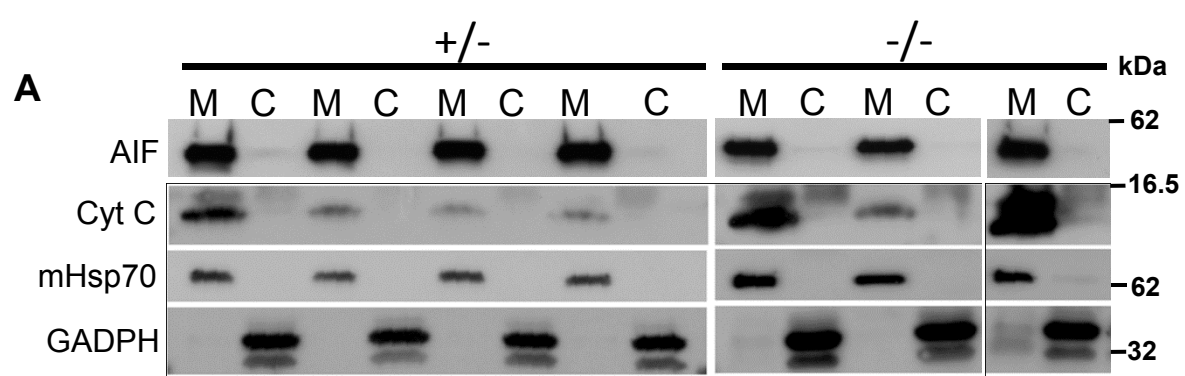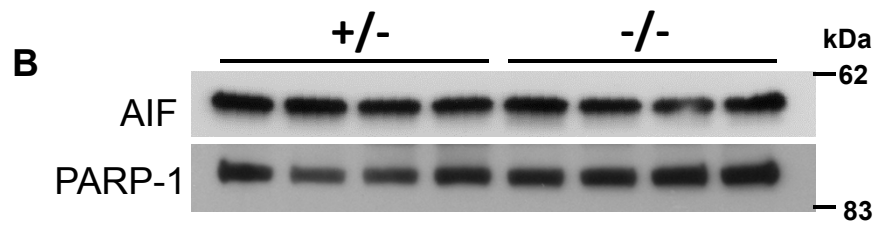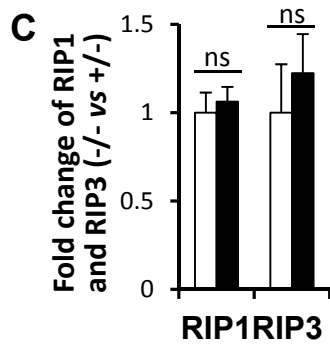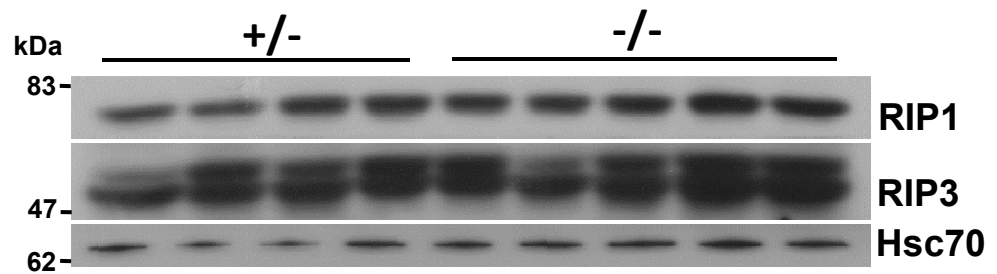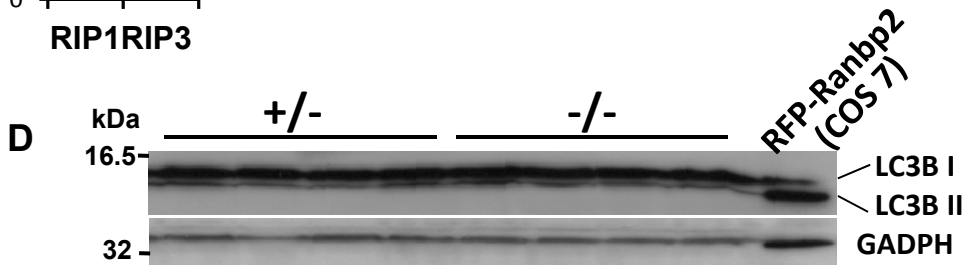

Supplement: Figure S8 — Lack of activation of canonical apoptotic, necroptotic and macroautophagic death upon ablation of Ranbp2 in cones at P13. (A) Subcellular fractionations of mitochondria (M) and cytosol (C) of retinal homogenates shows that there are no traces of AIF and cytochrome C (cyt C) release from the M to C fraction in either HRGP-cre:Ranbp2 +/− (+/−) or HRGP-cre:Ranbp2−/− (−/−) mice . GAPDH is a loading control. mHsp70 and GADPH are mitochondrial and cytosolic markers, respectively. (B) The levels of the apoptosis-inducing factor (AIF) are not changed in the nuclear-enriched fraction of either genotype . (C) The levels of receptor interacting proteins 1 and 3 (RIP1 and RIP3) remain unchanged between HRGP-cre:Ranbp2 +/− (+/−) and HRGP-cre:Ranbp2−/− (−/−) mice. Graph on the left is a quantification of immunoblot on the right. Hsc70 is a loading control. (D) The ablation of Ranbp2 in cone photoreceptors does not cause the generation of autophagosomal LC3B II, whereas ectopic expression of Ranbp2 in COS7 cells produces LC3B II (control). GAPDH is a loading control. Legend: mHsp70, mitochondrial heat shock protein 70; Hsc 70, cytosolic heat shock protein 70; GADPH, glyceraldehyde 3-phosphate dehydrogenase, LC3B II, autophagosomal membrane-associated light chain 3 II isoform; LC3B I, cytosolic light chain 3 II isoform; +/−, HRGP-cre:Ranbp2 +/−, −/−, HRGP-cre:Ranbp2−/−; Data shown represent the mean ± SD, n = 3–5; ns, not significant (p>0.05). (PDF) [file pgen.1003555.s008.pdf]

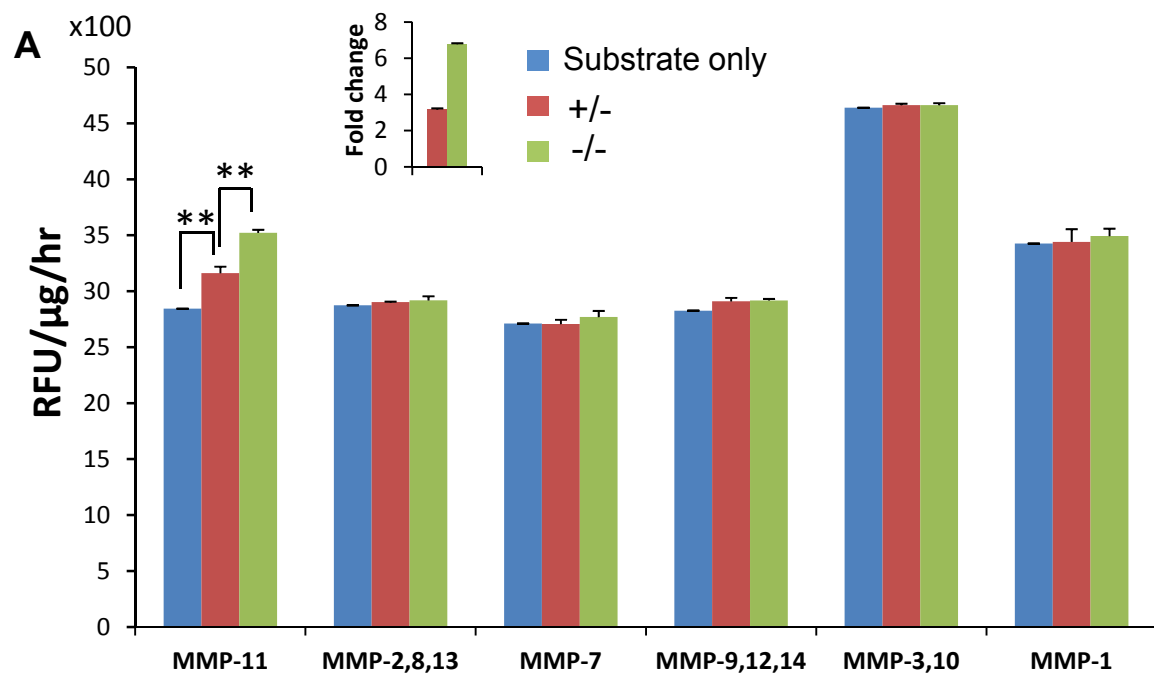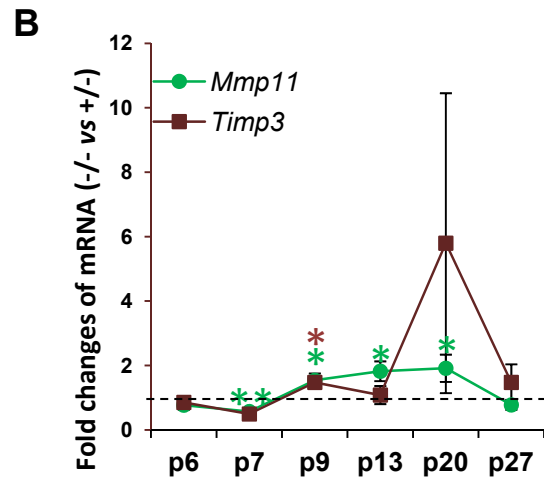

Supplement: Figure S9 — Selective activation and up-regulation of MMP11 upon ablation of Ranbp2 in cone photoreceptors. (A) Screening for activation of MMPs upon ablation of Ranbp2 in cones at P20. There is a selective activation of MMP11, but not of any other MMPs tested. In inset graph, the activity of MMP11 was normalized to control reactions without retinal extracts. HRGP-cre:Ranbp2−/− (−/−) compared to HRGP-cre:Ranbp2 +/− (+/−) mice have ∼3-fold increase of MMP11 activity. (B) qRT-PCR shows the transient down-modulation of Mmp11 at P7 followed by its sustained up-regulation until P20 in HRGP-cre:Ranbp2−/−. By contrast, the levels of Timp3 remain largely unchanged, except at P9, when there is a rise of its levels. Legends: Timp3, tissue inhibitor metalloproteinase 3; RFU, relative fluorescence units. Data shown represent the mean ± SD, n = 3–4. (PDF) [file pgen.1003555.s009.pdf]

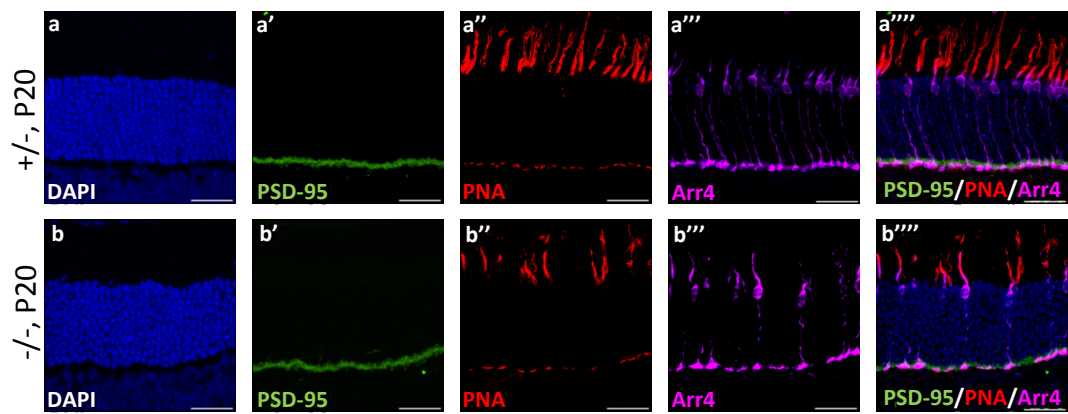

Supplement: Figure S10 — Localization of postsynaptic density 95 (PSD95) in HRGP-cre:Ranbp2−/− (−/−) compared to HRGP-cre:Ranbp2+/− (+/−). Immunolocalization of postsynaptic density marker, PSD-95, in retinas of +/− (a–a″″) and −/− mice (b–b″″) at P20. No changes of PSD95 were discerned between −/− and +/−. Legends: PSD-95, postsynaptic density protein 95, Arre4, cone arrestin 4, PNA, Peanut Agglutinin. Scale bars = 25 µm. (PDF) [file pgen.1003555.s010.pdf]

A

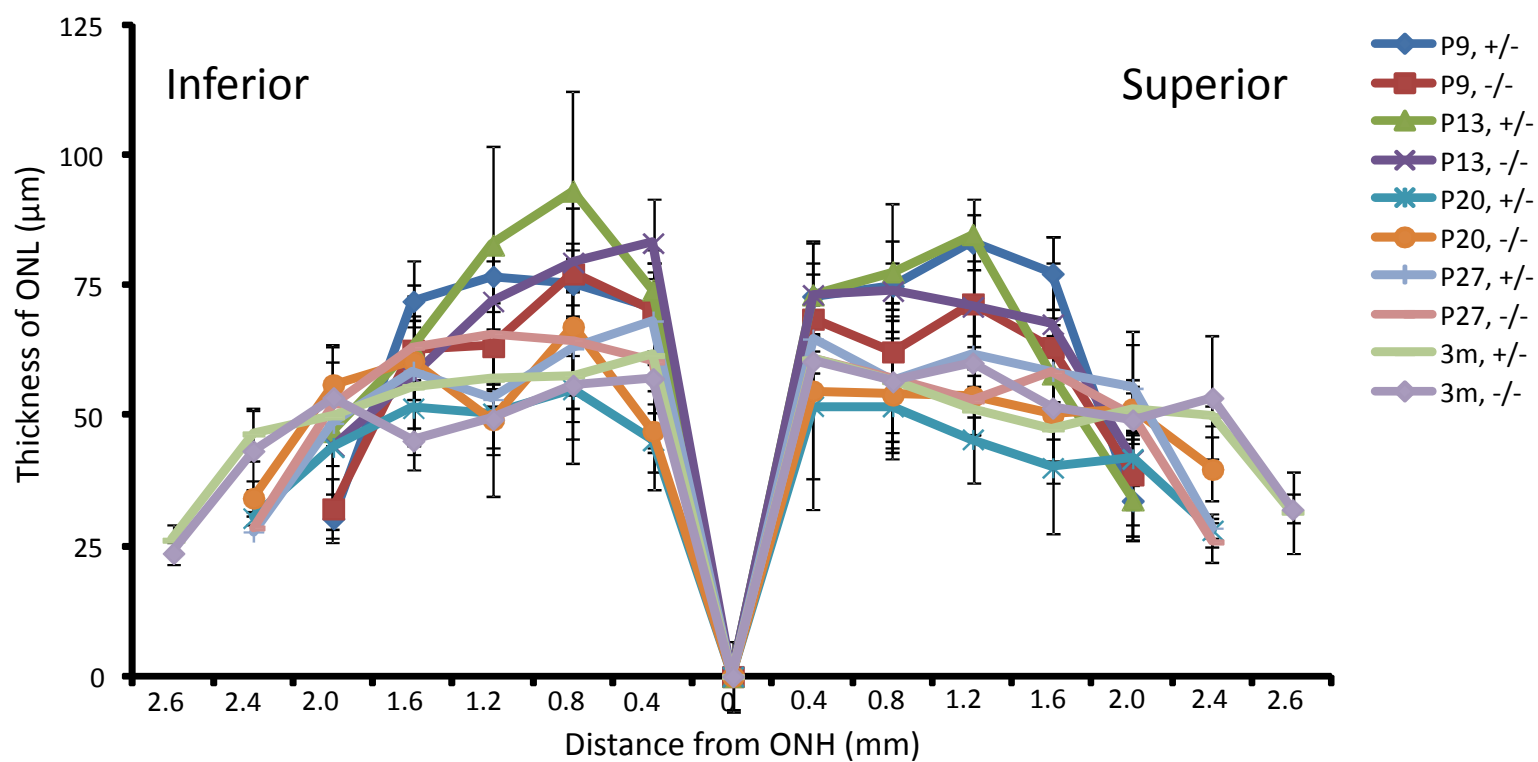

B

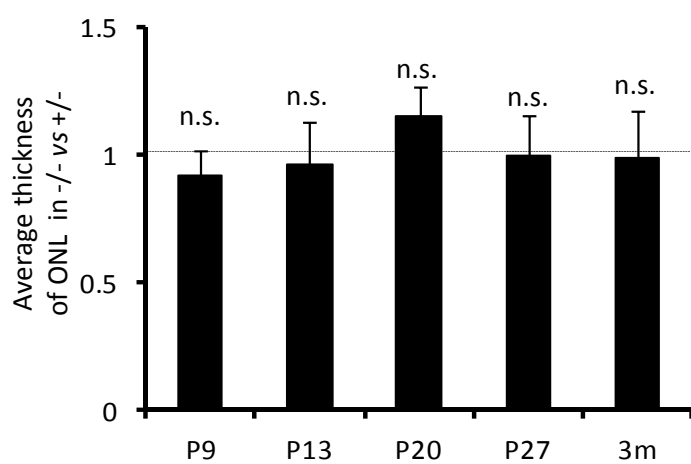

C

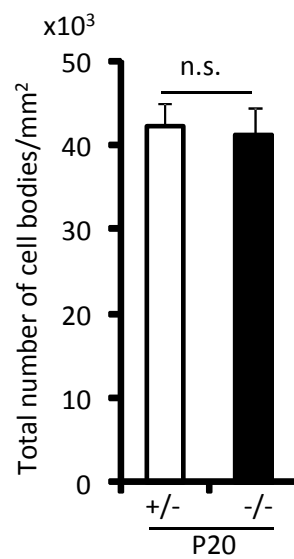

Supplement: Figure S11 — Absence of extended rod photoreceptor degeneration in HRGP-cre:Ranbp2−/− (−/−). (A) The quantification of the degeneration of rod photoreceptors by measuring the outer nuclear layer (ONL) thickness along the vertical meridian of the eye. There were no significant changes in ONL thickness between −/− and +/− from P9 to 3-month old mice. Plots demonstrate ONL thickness of at P9, P13, P20, P27 and 3-month of age. (B) Histograms of the average ratio of the ONL thickness between −/− and +/− from P9 to 3-month of age (p>0.05). (C) Comparison of photoreceptor cell body density in the ONL between −/− and +/− at P20. There were no significant differences. Nuclei in the ONL were counted and normalized per area from light microscopy images of methylene blue stained and semi-thin retinal sections. Legend: ONH, Optic nerve head, ONL, Outer nuclear layer. Data shown represent the mean ± SD, n = 3; n.s., not significant (p>0.05). (PDF) [file pgen.1003555.s011.pdf]
