# Supplementary material for: Distinct and Atypical Intrinsic and Extrinsic Cell Death Pathways between Photoreceptor Cell Types upon Specific Ablation of Ranbp2 in Cone Photoreceptors
Source: PLoS Genet. 2013 Jun 20;9(6):e1003555. doi: 10.1371/journal.pgen.1003555 (PMC3688534; doi:10.1371/journal.pgen.1003555)
Supplement: Table S1 — Designations and sequences of primers used for gene expression analyses by qRT-PCR. List of primers used for gene expression profiling of HRGP-cre:Ranbp2−/− and HRGP-cre:Ranbp2 +/− mice by qRT-PCR. (PDF) [file pgen.1003555.s012.pdf]

Table S1: Designations and sequences of primers used for gene expression analyses by qRT-PCR.

Significance	Gene symbol	Full name	Accession #	Forward	Reverse
Cone specific	*Opn1sw*	Opsin1(cone piGments), short-wave-sensitive	NM_007538.3	GCTGGACTTACGGCTTGTCACC	TGTGGCGTTGTGTTTGCTGC
	Opn1mw	Opsin1(cone piGments), medium-wave-sensitive	NM_008106.2	GTGAACAGACACTGGACCAC	CCACCTGGGAGCAATGTGATAA
	Pde6c	Phosphodiesterase 6C, cGMP-specific, cone, alpha prime	NM_033614.1	CTGGGTCGCTCATGTGAAGAAAG	CACAATCGGGACTGCCAGCAGA
	Pde6h	Phosphodiesterase 6H, cGMP-specific, cone, Gamma	NM_023898.1	TCCCAGGGACAGATGACCGTGATA	CAAGCAGAGGCAGACTCGACAGTTC
	Gnat2	Guanine nucleotide bindinG protein, alpha transducinG 2	NM_008141.1	GAAGATCATTCACCAGGATGGCTAC	CTGCCTAGTGTGGACATGGCTCT
	Osgep	O-sialoGlycoprotein endopeptidase	NM_133676.1	CGAGGCAAGAAGCTAGTCGAGCTG	TCAGGAGTACACTCTCCAGTGGCCA
Pan-photorecetpor	*Rcvrn*	Recoverin	NM_009038.2	GAGTACGTGATTGCTCTGCACATG	AGGAGTTTCACATCCTCAGGCTTG
Rod	*Rho*	Rhodopsin	NM_145383.1	GCTTCCCTACGCCAGTGTG	CAGTGGATTCTTGCCGCAG
Cone-survival	*pre-miR-124a*	MicroRNA 124 a2	NR_029814.1	ATCAAGATCAGAGACTCTGCT	TTCAAGTGCAGCCGTAGGCT
	Rncr3	retinal non-codinG RNA 3	NR_040709.1	CCAGCTAATAGATCTCCGAGCA	TGGTCAAGTGCCTTCCACATT
	Lhx2	LIM/homeobox protein 2	NM_010710.3	ATGCCACGCTGCAGACAGGGAC	TAGTCAAGTCTGTGAGGGTTGTA
	CoREST (Rcor1)	REST corepressor 1	NM_198023.2	AACAAGTCAGTTGTTCAGGTGAA	TGCTTCCCATTCTTGTAAAACTT
	Nrp1	Neuroplin 1	NM_008737.2	TGTACTGTGCCTGTTGGCACAATG	TTACACCATCCACAAAGT
Cone-transcription factors	*Trß2*	Tyroid hormone receptor beta (TRß2 isoform-specific)	NM_009380.3	ACTCCTGATGAAGGTGACAGA	ACAGAGGCGGGAAGAGCTCAGT
	Otx2	Orthodenticle homeobox 2	NM_144841.3	GTACCCAGACATCTTCATGAGGGA	CAGGCCTCACTTTGTTCTGACCTC
	Crx	Cone-rod homeobox containinG Gene	NM_007770.4	CACCAGGCTGTCCCATACTC	TCGCCCTACGATTCTTGAAC
MMPs	*Mmp11*	Matrix metalloproteinase 11	NM_008606.2	CATGCCGCCTTGGTCTGGGG	CGGGGCACGGGATTGTCCAC
	Col6α3	CollaGen type 6, alpha 3	NM_001243008.1	AGCATCCAGTTCAACCATCA	TCCCAGCATCTCTGGACA
	Col6α1	CollaGen type 6, alpha 1	NM_009933.4	AAAAGGGGCCCAGGCCAGTC	TCATGCGTGGCCTCACTCCG
	let7-c	microRNA let7c-1	NR_029728.1	TGTGTGCATCCGGGTTGAGGTA	AGTGTGCTCCAAGGAAAGCT
	Timp3	Tissue inhibitor of metalloproteinases 3	NM_011595.2	GCTGGAGCCTTGGGCACTGG	TCCCACCACTTTGGCCCGGA
Cell death	*Hif1a*	Hypoxia inducible factor 1, alpha subunit	NM_010431.2	AGTGGATTACCACAGCTGACCA	ATCCAAAGCTCTGAGTAATTCT
	Gfap	Glial fibrillary acidic protein	NM_001131020.1	GTCGGCCAGTTACCAGGAG	AGTAGATCCTGGTACTCCTGCA
	Casp3	Caspase-3	NM_009810.2	TGTACGCGCACAAGCTAGAATT	TGGAAAGTGGAGTCCAGGGAGA
	Casp7	Caspase-7	NM_007611.2	TGAACGACAGGGTGGCCAGGCA	TTGGTGAGCATGGACACCATA
	Casp8	Caspase-8	NM_009812.2	ACCGAGATCCTGTGAATGGAACC	TAAGAATGTCATCTCCTTGAGGA
	Casp9	Caspase-9	NM_015733.4	GGACAGTGACTCTGAGCCAGAT	AACTTGACACAGCATCCAGCTG
	Parp1	Poly [ADP-ribose] polymerase 1	NM_007415.2	ACAGGGATCCCATCTGGTGT	TGAGATTCACCTGAGCAATGTC
